# Galangin-loaded biomimetic dendritic cells membrane nanovaccine reprograms the ovarian cancer microenvironment *via* Stat3/IDO1/AhR axis to boost immunotherapy

**DOI:** 10.1016/j.mtbio.2026.102924

**Published:** 2026-02-10

**Authors:** Nuerbiye Aobulikasimu, Lele Fang, Aidiresi Maimaitiyiming, Dilimureti Kasimu, Adila Aipire, Weilan Wang, Zhongxiong Fan, Jinyao Li

**Affiliations:** a Xinjiang Key Laboratory of Biological Resources and Genetic Engineering, College of Life Science and Technology, Xinjiang University, Urumqi, 830017, China; b The First Affiliated Hospital of Xinjiang Medical University, Urumqi, 830017, China; c School of Pharmaceutical Sciences, Institute of Materia Medica, Xinjiang University, Urumqi, 830017, China

**Keywords:** Galangin, Dendritic cells vaccine, Anti-ovarian cancer, Tumor immune microenvironment, Stat3/IDO1/AhR signaling axis

## Abstract

Ovarian cancer, a highly lethal gynecological malignancy with high recurrence rates and low survival, poses significant treatment challenges. While immunotherapy, particularly dendritic cell (DC) vaccines, boosts immune responses, its clinical efficacy is limited by poor antigen presentation and weak lymph node targeting. To address this, we develop a novel biomimetic nanovaccine (GA-NPs@DCV) using cell membrane-coated nanoparticles that incorporate the natural anti-tumor agent galangin (GA) and ovarian tumor-associated antigens (TAAs). GA-NPs@DCV features the antigen-presenting functions of DCs and utilizes GA to induce tumor immunogenic cell death (ICD), which not only endows the vaccine with abundantly processed specific TAAs, but also ensures robust homing capability to lymph nodes and the tumor microenvironment. Interestingly, in OT-I/OT-II transgenic mice, GA-NPs@DCV enhances the proliferation of CD8^+^ and CD8^+^ IFN-γ^+^ cells to activate potent immune responses. Furthermore, a nano-DC vaccine carrying distinct antigens (NPs@DCV) not only inhibits tumor growth and activates systemic immune responses, but also effectively prevents tumor recurrence. Importantly, GA-NPs@DCV exerts potent anti-ovarian cancer effects by promoting immune activation, inhibiting immune evasion through the Stat3/IDO1/AhR signaling axis, and remodeling tumor immune microenvironment *via* regulation of the tryptophan metabolic pathway. Notably, GA-NPs@DCV also promotes the generation of tissue-resident memory T cells (T_RM_, CD8^+^CD103^+^ cells) within tumor tissue, effectively inducing long-term protective immunity. Overall, these findings identify GA-NPs@DCV as an effective personalized nanovaccine that can simultaneously deliver tumor antigens and the Stat3 inhibitor GA to the tumor microenvironment to exert potent antitumor effects, providing a promising immunotherapeutic strategy for ovarian cancer.

## Highlights

• Galangin (GA), as a Stat3 inhibitor, exhibits anti-ovarian cancer (OC) activity.
• GA-NPs@DCV is constructed to mimic APCs and home to lymph nodes and tumors.
• GA-NPs@DCV enhances antigen-specific CTL responses to boost anti-OC immunotherapy.
• GA-NPs@DCV targets Stat3/IDO1/AhR axis to remodel tumor immune microenvironment.
• GA-NPs@DCV promotes the generation of T_RM_ for long-term protection against OC.

## Graphical abstract

**Figure:**
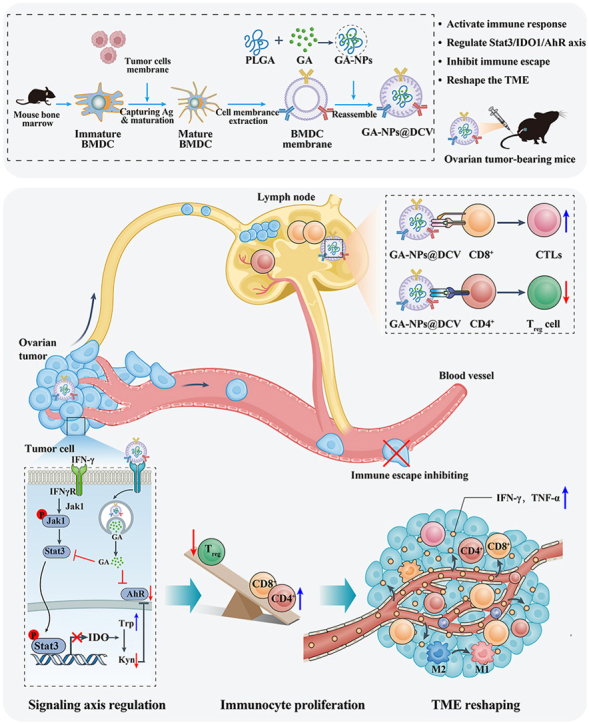

## 1 Introduction

Due to diagnostic limitations and subtle symptoms, ovarian cancer (OC) is often detected at advanced stages, thus increasing recurrence and metastasis risks [1]. In recent years, immunotherapy has emerged as a powerful approach to restrain relapse, yet the inherently low immunogenicity of OC curtails patient responsiveness [2]. As a critical form of immunotherapy, dendritic cells (DC)-based cancer vaccines, which are capable of inducing antigen-specific immunogenic responses, have made significant progress in recent years in enhancing anti-tumor immunity against poorly immunogenic tumors [[3], [4], [5]]. Nevertheless, the clinical efficacy of DC vaccines is still hindered by the inefficient presentation of tumor-associated antigens (TAAs) *via* MHC I molecules and the low efficiency of lymph node homing [6]. To address these limitations, biomimetic vaccines based on cell membrane systems, particularly those utilizing DC membranes for surface modification, have shown remarkable advantages. By preserving the surface protein profile of native DCs, this strategy not only enhances antigen presentation but also improves tissue penetration and retention *in vivo*, thereby overcoming the biological barriers associated with conventional DC vaccines [[7], [8], [9]]. Additionally, the incorporation of nanocarrier systems enhances the antigen-loading potential and protective profile of biomimetic nanovaccines, allowing for the precise co-delivery and controlled release of tumor antigens and immunomodulators to ensure the biomimetic nanovaccines stability and continuous immune activation [10,11]. In summary, the integration of biomimetic coating technology with the advantages of nanocarriers represents a highly promising new strategy for efficient immunotherapy in ovarian.

DCs, as the most efficient antigen-presenting cells (APCs), activate naïve T cells and induce cytotoxic T lymphocytes (CTLs), thereby driving anti-tumor immunity [12,13]. Consistently, increased DC infiltration has been associated with prolonged survival in cancer patients [14]. However, the tumor microenvironment (TME) may impede the maturation and function of DCs. Thus, exploring some novel and effective strategies to enhance DC functionality within the TME is crucial for the development of more effective cancer immunotherapies. Dysregulation of Stat signaling profoundly impacts anti-tumor responses in the TME. Specifically, Stat3 activation promotes pro-tumorigenic factors, suppresses chemokine expression, and hinders DC maturation and antigen transport, thereby excluding T cells from effective immune engagement [15]. Furthermore, phosphorylation following Stat3 activation causes nuclear translocation, activating the expression of downstream genes such as indoleamine 2,3-dioxygenase 1 (IDO1) [16]. In solid tumors, the endogenous IDO1 is highly expressed and contributes to the immunosuppressive tumor microenvironment. Overexpressed IDO1 can catalyze the decomposition of tryptophan (Trp) into kynurenine (Kyn), thereby activating the aryl hydrocarbon receptor (AhR) and further exacerbating tumor immunosuppression and immune evasion [17,18]. Trp depletion diminishes the metabolic activity of CTL, while elevated Kyn recruits more regulatory T cells (Tregs), ultimately leading to tumor immune escape and immunotherapy failure [19,20]. Based on this, we hypothesize that developing novel drugs that regulate the Stat3/IDO1/AhR signaling axis within the TME, in conjunction with existing therapeutic modalities, will provide new insights for OC treatment.

Fortunately, natural products, particularly plant-derived compounds, constitute approximately 60% of current anticancer drugs, underscoring ongoing research into more efficacious and safer treatments from natural sources [21]. Galangin (GA), a natural flavonoid, exhibits significant potential against various malignancies, including ovarian cancer [22]. Mechanistically, studies have revealed that GA inhibits the Jak/Stat3 pathway and Myc activation, reducing PD-L1 expression to enhance T-cell-mediated tumor killing [23,24]. Our previous work has revealed that GA directly binds Stat3, suppressing the Stat3/IDO1/AhR axis and inducing immunogenic cell death (ICD), positioning it as a promising anticancer candidate. However, GA's low solubility, slow absorption, and rapid metabolism hinder its clinical application [25]. Recently, with the rapid advancement of nanomedicine, nanoparticles (NPs) have emerged as a promising strategy to leverage the enhanced permeability and retention (EPR) effect for improved tumor accumulation [26]. Typically, poly (lactic-co-glycolic acid) (PLGA) exhibits excellent biocompatibility, biosafety, and sustained release characteristics. Furthermore, PLGA nanoparticles possess inherent capabilities for antigen delivery and adjuvanticity, enhancing T cell responses and activating antigen-presenting cells to augment cancer immunotherapy [27,28], making them an ideal carrier for GA. Therefore, functionalizing PLGA nanoparticles with DC membranes is expected to be a promising strategy to improve therapeutic efficacy *via* integrating APC activation, enhanced T cell responses, and direct tumor targeting.

**Scheme 1:**
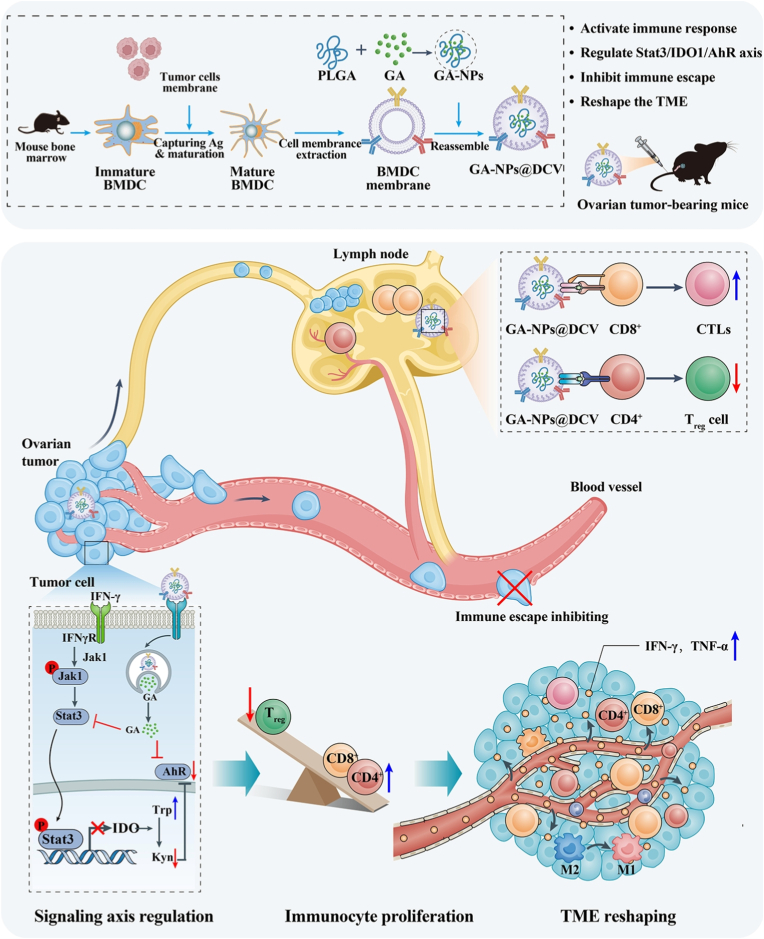
Schematic illustration of the construction and mechanism of the biomimetic nano-DC vaccine (GA-NPs@DCV) in ovarian cancer immunotherapy.

While direct conjugation of tumor-specific antigens onto nanoparticles is a common strategy, its efficacy is contingent upon the subsequent uptake and processing by host DCs, a process that can be inefficient and rate-limiting, especially within the immunosuppressive tumor microenvironment. To address this, a tumor antigen-specific biomimetic nanovaccine (GA-NPs@DCV) is constructed by using cell membrane-coated nanoparticles to incorporate the natural anti-tumor agent GA and ovarian tumor-associated antigens (TAAs), as illustrated in Scheme 1. The GA-NPs@DCV is characterized by antigen-presenting functions and GA-induced ICD in tumor cells. This design not only endows it with an abundance of processed specific TAAs, but also confers a robust capability of homing to lymph nodes and the tumor microenvironment. By inhibiting the Stat3/IDO1/AhR signaling pathway, GA-NPs@DCV blocks immune evasion and further reshapes the immune microenvironment through tryptophan metabolism, promoting the proliferation and activation of antitumor immune cells, thereby exerting potent antitumor effects. Thus, GA-NPs@DCV provides a promising and effective therapeutic strategy for OC treatment.

## 2 Materials and methods

### 2.1 Materials

3,5,7-Trihydroxyflavone (Galangin, GA) and NLG919 were purchased from Macklin Biotechnology (Shanghai, China). Carboxyl-terminated 50:50 (PLGA) was supported from Meilun Biotechnology (Dalian, China). The polyvinyl alcohol (PVA), ConA, tryptophan, kynurenine, and β-mercaptoethanol were all obtained from Sigma-Aldrich (Saint Louis, USA). The bicinchoninic acid (BCA) kit, protease and phosphatase inhibitor cocktail, 5 × SDS-PAGE sample loading buffer, hoechst 33342, DiD, 3-(4,5-dimethylthiazol-2-yl)-2,5-diphenyltetrazolium bromide (MTT) were all purchased from Beyotime Biotechnology (Jiangsu, China). The lipopolysaccharide (LPS), eBioscience™ CFSE, PMSF, murine recombinant granulocyte/macrophage colony-stimulating factor (GM-CSF), recombinant Murine interferon-γ (IFN-γ), culture medium and fetal bovine serum (FBS) were all obtained from Thermo Fisher Scientific (MA, USA). Mouse IFN-γ and TNF-α ELISA kit were purchased from R&D systems (Minneapolis, USA). Antibodies for western blotting and flow cytometry were listed in Tables S1–S2.

### 2.2 Cells culture

The murine ovarian cancer cell line ID8 (RRID: CVCL_IU14) was obtained from iCell Bioscience (Shanghai, China) and authenticated to be contamination-free *via* STR genotyping and mycoplasma detection reports. The cell lines Caov-3 (RRID: CVCL_0201), Skov-3 (RRID: CVCL_0532), RAW 264.7 (RRID: CVCL_0493), and B16 (RRID: CVCL_F936) were preserved in liquid nitrogen tanks in the Xinjiang Key Laboratory of Biological Resources and Genetic Engineering. Cells were cultured in DMEM supplemented with 10% FBS and 1% penicillin-streptomycin at 37 °C in a 5% CO_2_ atmosphere. Primary mouse BMDCs derived from C57BL/6 mice were induced by GM-CSF and cultured as our previous description [29]. Briefly, bone marrow cells were isolated from the femurs and tibias of C57BL/6 mice and cultured in RPMI-1640 plates supplemented with 10% fetal bovine serum, 1% penicillin-streptomycin, 50 μM β-mercaptoethanol, and 20 ng/mL GM-CSF. Half of the medium was replaced on days 2 and 5, and the medium was completely replaced on day 3. On day 6, BMDCs were harvested for further experiments.

### 2.3 Cell viability assay

To assess the cytotoxicity of GA-NPs@DCV in ID8, RAW.267.4, 293T cells and BMDCs, cell viability was measured using the MTT and CCK8 kit 24 h after administration with various formulations according to the standard protocol.

### 2.4 Animals

The OT-I/OT-II transgenic C57BL/6 mice, which express T cell receptors specific for the ovalbumin peptides OVA257-264 and OVA323-339 respectively, were purchased from the Jackson Laboratory (Maine, USA). Wild-type C57BL/6 mice (4–6 weeks old) were obtained from the Animal Experimental Center of Xinjiang Medical University (Urumqi, China). All mice were housed in the animal facility of Xinjiang University. The experimental protocols were approved by the Committee on the Ethics of Animal Experiments of Xinjiang Key Laboratory of Biological Resources and Genetic Engineering (XJUAE-2024-015), in accordance with the NIH (National Research Council) Guide for the Care and Use of Laboratory Animals.

### 2.5 LiP-MS assay

To identify proteins that directly bind to GA, LiP-MS assay was performed by SpecAlly Life Technology (Wuhan, China) following a protocol previously reported [30]. In brief, ID8 cells were lysed and centrifuged at 12000 rpm for 5 min at 4 °C. Protein concentrations were determined using a BCA assay kit (Beyotime Biotechnology). Total protein (500 μg) was incubated with 30 μM of GA or DMSO for 10 min at 25 °C (n = 3). Proteinase K (5 μg, Sigma-Aldrich) was added to each sample, followed by incubation for an additional 10 min. The reaction was terminated by heating at 98 °C for 3 min. Samples underwent limited proteolysis to generate structure-specific protein fragments. Trypsin (Promega) digestion was performed at an enzyme-to-substrate ratio of 1:50 at 37 °C overnight, and the reaction was stopped by adding trifluoroacetic acid. Subsequently, the supernatants were immediately analyzed using an using UltiMate3000RSLCnano nanoliter liquid phase (Thermo) in series with timsTOFPro mass spectrometer (Bruker). The obtained raw data were processed through rigorous computational workflow, including sample quality control, peptide label-free quantitation, and screening for GA-binding proteins. Ultimately, 66 peptides corresponding to 61 candidate proteins were identified and further validated by Western blotting.

### 2.6 Cellular thermal shift assay (CETSA)

To further confirm whether GA directly binds to Stat3, a Cellular Thermal Shift Assay (CETSA) was performed. This assay is based on the principle that ligand binding modifies a target protein's thermal stability, thereby reducing its propensity for denaturation compared to the unbound protein at the elevated temperatures. ID8 cells were lysed and centrifuged, and the supernatant was collected. The lysate was divided into two aliquots: the experimental group was treated with 30 μM GA, while the control group received an equal volume of DMSO. Both aliquots were incubated on an ice shaker for 1 h. Subsequently, the samples were aliquoted and heated at various temperatures (37, 45, 50, 60, 70, 80, and 90 °C) for 3 min. After cooling and centrifugation, the soluble protein fractions were analyzed by Western blotting to assess changes in Stat3 thermal stability.

### 2.7 Detection of ICD biomarkers

Calreticulin (CRT) and High Mobility Group Box 1 (HMGB1) were evaluated using immunofluorescent techniques. ID8 cells, at a density of 8 × 10^4^ per well, were seeded onto glass-bottom dishes for 24 h. Subsequently, the cells were treated with media supplemented with GA (30 μM) for 6 h. Surface CRT exposure and HMGB1 expression were detected by Confocal Laser Scanning Microscopy (Nikon, AIR HD25, Japan) using antibodies against CRT (Beyotime, AF1666) and HMGB1 (Abcam, ab79823).

### 2.8 ID8 Co-culture with spleen lymphocytes

To evaluate the antitumor effects mediated by enhanced immune responses *in vitro*, tumor cells and splenic lymphocytes were co-cultured [31]. Specifically, ID8 cells were seeded into 24-well plates at a density of 5 × 10^4^ cells per well overnight. Splenic lymphocytes were isolated from the spleen of healthy C57BL/6 mice, and then added to the plates at an effector-to-target ratio of 10:1 (lymphocytes:ID8). The co-cultured cells were treated with the different formulations for 72 h to assess immune cell proliferation.

### 2.9 Construction of GA-NPs

Galangin-loaded PLGA nanoparticles were prepared by emulsion solvent evaporation method [32]. Specifically, 4 mg of GA and 10 mg of PLGA were co-dissolved in 1 mL dichloromethane to form the organic phase. This organic solution was added to an aqueous solution containing 3% PVA and emulsified *via* probe sonication to form an oil-in-water (O/W) emulsion. The emulsion was sonicated at 100 W with a 5 s on/off cycle for 10 min to reduce droplet size and form nanoparticles. Dichloromethane was allowed to evaporate under magnetic stirring for 6 h, and the resulting GA-loaded PLGA nanoparticles (GA-NPs) were collected by high-speed centrifugation.

### 2.10 Isolation of ID8 tumor cell membrane

Extraction of ID8 cell membranes hypotonic lysis, repeated freeze-thawing, and gradient centrifugation, as previously reported [33,34]. Briefly, ID8 cell harvested and lysed in 2 mL of hypotonic solution on ice for 1 h. Then the tumor cells were frozen-thawing in liquid nitrogen at least six cycles, followed by ultrasonic disruption at 100 W with 5 s on/off cycles for 10 min. The obtained homogenate was centrifuged at 4 °C and 2000 rpm for 10 min, and the resulting supernatant was further centrifuged at 15,000 rpm for 30 min at 4 °C. The obtained precipitates were washed three times with deionized water. The protein concentration of ID8 cell membranes was measured using BSA assay, and the membranes were preserved in PBS at −80 °C for subsequent experiments.

### 2.11 Preparation of GA-NPs@DCV

The galangin-loaded biomimetic dendritic cell-based nanovaccines (GA-NPs@DCV) were constructed by cell membrane-coated nanoparticles technology [35]. First, to evaluate the maturation and antigen presentation of DCs pulsed with tumor cell membrane, BMDCs on the 6th day were collected and incubated with tumor cell membranes at a protein concentration of 50 μg/mL for 12 h. Subsequently, the mixture was centrifuged at 2000 rpm for 10 min to pellet the mature DCs, while the supernatant containing unbound tumor cell membranes was discarded. The cell pellet was then washed three times with cold PBS to remove any residual tumor membrane fragments. Finally, purified DC membranes (DCM) carrying tumor antigens were obtained *via* hypotonic lysis and ultrasonic fragmentation, repeated freeze-thawing, and gradient centrifugation, as detailed in Section 2.10.

The weight ratio of DCM to GA-NPs cores was optimized to achieve the best physicochemical properties and biological functionality. In a pilot study, we systematically compared ratios of 1:2, 1:1, and 2:1. The formulations were evaluated based on nanoparticle size, polydispersity, morphology, and targeting efficiency. The 1:1 wt ratio was selected as the optimal formulation, as it demonstrated the most uniform size distribution and superior targeting capabilities, effectively balancing physical stability with functional performance (Fig. S1). To prepare the DC membrane-coated nanoparticles, the obtained DCM were mixed with PLGA nanoparticulate cores at a weight ratio of 1:1, sonicated at 50 W with 3 s on/off cycles for 5 min, and physically extruded through polycarbonate membranes with pore sizes of 800 nm and 450 nm six times each. Excess DCM and nanoparticles were removed by low-speed centrifugation (3000 rpm), and the supernatant was further centrifuged at high-speed centrifugation (15000 rpm) to collect the precipitation (GA-NPs@DCV), which was stored in PBS at −80 °C for future experiments.

### 2.12 Characterization of GA-NPs@DCV

The size and zeta potentials of GA-NPs@DCV were determined using a Malvern Laser Particle Size Zeta Potential Analyzer (Zetasizer Nano ZS90, UK), and the morphology was examined using Transmission Electron Microscopy (TEM, JEOL JEM 2100 F). The total protein contents of DCM, NPs@DCV, GA-NPs@DCV were identified by Coomassie blue staining and Western blotting. To preliminarily evaluate the biosafety of the GA-NPs@DCV vaccine, a hemolysis assay was conducted. Mouse red blood cells were treated with GA-NPs@DCV (50, 100, 200, 500 μg/mL), GA, and deionized water, followed by incubation and centrifugation to evaluate the extent of hemolysis. The encapsulation efficiency and loading capacity of GA-NPs@DCV were measured by high performance liquid chromatography (HPLC, Agilent LC-1260) under the following chromatographic conditions: chromatographic column: Poroshell 120 EC-C18 (4.6 × 150 mm, 4 μm), mobile phase: 1% formic acid/acetonitrile (20:80, v/v), flow rate: 1.0 mL/min, column temperature: 25 °C, and detection wavelength: 280 nm. To investigate the *in vitro* GA release profile, GA-NPs@DCV and GA-NPs were in dialysis membranes (MWCO 3500 Da), and incubated in PBS. At predetermined time points, aliquots of the release medium were collected and analyzed by HPLC. The drug loading (DL) and encapsulation efficiency (EE) were calculated using the following equations.

DL(%)=(MassofGAinGA‐NPs@DCV/TotalMassofGA‐NPs@DCV)×100%

EE(%)=(MassofGAinGA‐NPs@DCV/TotalMassofGA)×100%

### 2.13 In vitro antigen-specific T-cell responses

To evaluate the antigen-specific binding of GA-NPs@DCV to T cells, splenic lymphocytes isolated from healthy C57BL/6 mice and incubated with DID-labeled GA-NPs@DCV for 1 h at 4 °C. Following incubation, cells were stained with a fluorescently-labeled anti-CD3 antibody. The co-localization of the T lymphocyte surface marker CD3 and DID was assessed using immunofluorescence and Confocal Laser Scanning Microscopy (CLSM). The DID fluorescence intensity within the CD3^+^ T cells population was further assessed by flow cytometry.

Additionally, splenic lymphocytes from OT-I/OT-Ⅱ mice were stained with CFSE, and seeded into 24-well plates at a density of 1 × 10^5^ cells per well. The GA-NPs@DCV used in the antigen-specificity assay was constructed through a specific process: BMDCs derived from OT-I/OT-II mice were pulsed with OVA257-264 and OVA323-339 (OT-I/OT-II peptides) respectively, rather than ID8 membrane. Following maturation, BMDCs membranes loaded with OT-I/OT-II peptides were harvested to form the nano DC vaccines. These vaccines were then used to treat splenic lymphocytes from OT-I/OT-II mice for 72 h. Flow cytometry was utilized to analyze the proliferation of CD8^+^ T and CD4^+^ T cells among splenic lymphocytes treated with DC+ OT-I/OT-II peptides, DCM, NPs@DCV, and GA-NPs@DCV, thereby evaluating the antigen-specific efficacy.

### 2.14 Acute toxicity Assessment of GA-NPs@DCV

To evaluate the *in vivo* biosafety of GA-NPs@DCV, an acute toxicity test was performed in healthy female C57BL/6 mice (6-8 weeks old). The mice were randomly divided into four groups (n = 8 per group): a control group (intravenously injected with saline) and three treatment groups that received a single intravenous injection of GA-NPs@DCV at doses of 20, 50, and 100 mg/kg, respectively. Over a 14-day observation period, the mice were monitored for mortality, changes in body weight, and any signs of behavioral abnormalities. On day 14, blood was collected for serum biochemical analysis of liver (ALT, AST), kidney (BUN, Cr), and cardiac (CK-MB) function. Subsequently, major organs including the heart, liver, spleen, lung, kidney, and ovaries were harvested for histopathological examination *via* H&E staining.

### 2.15 In vivo targeting Assessment

To investigate the *in vivo* distribution of GA-NPs@DCV, ICG-labeled GA-NPs@DCV and ICG were injected into ID8 tumor-bearing mice for tracking with the IVIS Lumina X5 system (PerkinElmer, USA). This technique utilizes ICG as a near-infrared (NIR) fluorophore. Upon excitation at approximately 780 nm, ICG emits fluorescence in the NIR spectrum (∼830 nm), which penetrates biological tissue effectively with minimal autofluorescence interference. Drug accumulation was monitored at predetermined time points. Following *in vivo* imaging, major organs and tumor specimens were excised for *ex vivo* fluorescence imaging analysis.

### 2.16 Anti-melanoma efficacy

In light of the excellent immune activation and targeted homing capabilities of GA-NPs@DCV, it draws our attention to its anti-tumor effect *via* initiating specific immune response. Therefore, we constructed a melanoma model as a proof-of-concept to further validate the specific antigen presentation of the nano-DC vaccine and its ability to elicit an antigen-specific immune response. To establish the B16-OVA tumor model, B16-OVA cells (1 × 10^5^) were subcutaneously injected into the right flank of female C57BL/6 mice (n = 7). Six days after inoculation of tumor cells, tumor-bearing mice were immunized *via* tail vein injection with NPs@DCV (10 mg/kg) carrying different antigens (B16, B16-OVA, OVA). The tumor volume and body weight were monitored. Following the completion of six immunotherapy sessions, flow cytometry was utilized to examine immune cell subsets, including T cells, DCs, and TAMs in the spleen and lymph nodes. Concurrently, splenocytes were re-stimulated with different antigens to assess antigen-specific responses. Serum samples was analyzed to determine the levels of immune cytokines by ELISA.

### 2.17 Therapy in ovarian cancer model

Ovarian cancer ID8 cells (1 × 10^6^) were inoculated subcutaneously into the right flank of female C57BL/6 mice. Animals were randomly divided into six groups (n = 7): PBS, GA (25 mg/kg), GA-NPs, PLGA, NPs@DCV, and GA-NPs@DCV (10 mg/kg). The therapeutic regimen was initiated at a tumor volume reached approximately 100 mm^3^ to model advanced-stage ovarian cancer and evaluate the nano-vaccine's therapeutic potential [36]. The vaccinations were administered every four days to align with the kinetics of the adaptive immune response, ensuring sufficient T-cell priming and expansion. Body weight and tumor size of mice in each group were recorded. On the 22nd day, the mice were sacrificed. Tumor tissues were dissected and subjected to Tunel staining and immunofluorescence analysis to detect apoptosis and immune cell infiltration. H&E staining was used to evaluate morphological changes in major organs (heart, liver, spleen, lung, and kidney) and tumor tissues. Serum levels of TNF-α and IFN-γ were measured by ELISA. Additionally, serum biochemical analysis was performed to assess liver function (ALT, AST), kidney function (BUN, Cr), and cardiac function (CK-MB). HPLC was utilized to determine the concentrations of tryptophan and kynurenine in tumor tissues. Flow cytometry was used to assess the relative abundance of various immune cell subsets (including T cells, Tregs, and T_RM_ cells) within the tumor tissues.

### 2.18 Immunofluorescence staining

The cells were fixed using 4% paraformaldehyde (PFA) for a duration of 10 min. To permeabilize the cells, a 0.2% solution of Triton X-100 was applied for 15 min, followed by a 30 min incubation with 5% bovine serum albumin (BSA) to block nonspecific binding sites. The cells were then incubated overnight at 4 °C with primary antibodies. Subsequently, the cells were stained with secondary antibodies for 1 h at 25 °C. To visualize the cell nuclei, Hoechst 33342 was added for 10 min at 25 °C. Finally, visualization of the cells was performed using a Confocal Laser Scanning Microscope (Nikon, AIR HD25, Japan).

### 2.19 Flow cytometric analysis

Spleens were harvested, washed with PBS, and homogenized through a 200-mesh copper mesh. The resulting cell suspension was treated with red blood cell lysis buffer and centrifuged (1200 rpm, 5 min). Tumor tissues were minced and digested in RPMI-1640 medium containing hyaluronidase, DNase I, and collagenase I at 37 °C for 1 h. The digest was then filtered through a 200-mesh copper mesh, washed with PBS, and centrifuged. The cell suspension was stained with appropriate fluorescently-conjugated antibodies (Table S1) and analyzed for immune cell subsets using flow cytometry.

### 2.20 Western blotting experiment

Proteins were extracted from cells and tissue samples using RIPA lysis buffer containing phosphatase and protease inhibitors, incubated for 30 min, and then centrifuged (12000 rpm, 5 min, 4 °C). The supernatant was collected for the protein concentration using an BCA protein assay kit, then prepared by heating at 100 °C for 10 min in loading buffer. Following this, the protein samples were separated with SDS-PAGE and transferred onto a 0.45 μm PVDF membrane (Millipore Corporation, MA, USA). After blocking with 5% BSA, the membrane was sequentially incubated with primary antibodies and secondary antibody (Table S2). All bands were visualized using ECL detection, and images were quantified by ImageJ 1.54h software (NIH, Bethesda, MD, USA).

### 2.21 Statistical analysis

All data were presented as means ± SD using GraphPad (Prism 9.5.1). The statistical significance between two groups or more than three groups was evaluated using Student's t-test or one-way ANOVA, respectively. All experiments were independently performed at least three times. The levels of significant differences were indicated by *p* < 0.05. (∗*p* < 0.05, ∗∗*p* < 0.01, and ∗∗∗*p* < 0.001).

## 3 Results and discussion

### 3.1 Anti-tumor ativity of GA by inhibiting stat3/IDO1/AhR signaling axis through directly binding to Stat3

**Fig. 1:**
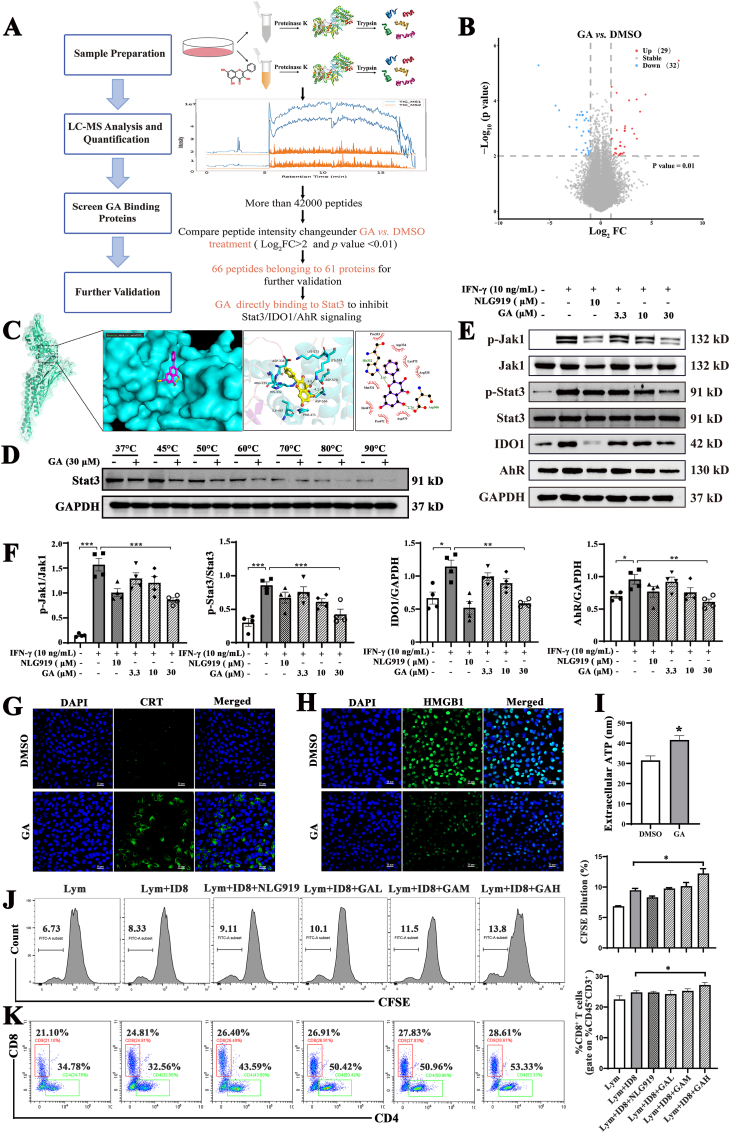
GA directly binds to Stat3 and causes ICD of tumor cells by inhibiting Stat3/IDO1/AhR signaling axis. A) The flowchart of the LiP-MS assay. Freshly prepared whole-cell lysates of ID8 were exposed to GA, followed by digestion with proteinase K and trypsin, and then subjected to mass spectrometry (MS) analysis. The binding of GA inhibited protease digestion, resulting in differences in the MS peptide profile. B) Visualization of LiP-MS assay results using volcano plots (Log_2_FC > 2 and *p* value < 0.01). C) Molecular docking assessing the binding mode of GA with Stat3. D) Cellular Thermal Shift Assay (CETSA) method was used to explore the thermal instability of Stat3 and GA interaction across a temperature gradient ranging from 37 to 90 °C. E, F) Representative western blots analysis of Jak1, p-Jak1, Stat3, p-Stat3, IDO1, and AhR after treatment with the different dose of GA and NLG919 in IFN-γ-induced ID8 cells (n = 4). NLG919 is an IDO1 inhibitor and serves as a positive control. G, H) The immunofluorescence staining of CRT and HMGBl in ID8 cells after administration with GA, scale bar = 25 μm. I) The extracellular ATP levels after administration with GA in ID8 cells (n = 6). J) The T cell proliferation was assessed by flow cytometry in the CFSE-stained spleen lymphocytes from healthy C57 mice co-cultured with ID8 cells in target ratios of 1:10, meanwhile treatment with different formations for 72 h. K) The CD8^+^ T cells was assessed by flow cytometry in the co-cultured system (n = 6). Data are presented as mean ± SD and *p*-values <0.05 were considered significant (∗*p* < 0.05, ∗∗*p* < 0.01, ∗∗∗*p* < 0.001).

A limited proteolysis-mass spectrometry (LiP-MS) assay was utilized to identify proteins that directly bind to GA (Fig. 1A). As shown in Figs. 1B and 66 peptides belonging to 61 proteins were identified. Among them, the transcription factor Stat3 demonstrated a high binding affinity for GA. It has also been reported that GA displays anti-cancer activity by suppressing Jak1/Stat3 to enhance immune response activation [24,25]. Molecular docking revealed that GA binds effectively to Stat3 *via* hydrogen bonds with LYS574, ARG335, and ASP556, indicating the formation of a stable complex (Fig. 1C). CETSA is predominantly utilized to confirm interactions between drugs and their target proteins [37,38]. CETSA results revealed that GA significantly promoted the degradation of Stat3 protein at all tested temperatures (Fig. 1D). As shown in Fig. S2, GA treatment significantly reduced the Tm of Stat3 from 68.86 °C to 60.20 °C (ΔTm = −8.66 °C, *p* < 0.05) compared to the control, indicating that GA binding decreases the thermal stability of Stat3. Therefore, the observed thermal instability provides compelling evidence for a direct and disruptive interaction between GA and Stat3, which underpins its downstream inhibitory effects.

Additionally, studies have confirmed that Stat3 enhances the expression of IDO1 by binding to the cis-regulatory region of IDO1, resulting in the elevation of kynurenine and thus aryl hydrocarbon receptor (AhR) activation, which further aggravates tumor immunosuppression and tumor immune evasion [16,18,39]. Bioinformatics analysis shown in Fig. S3 indicated that IDO1 was aberrantly highly expressed in OC patients, and the resulting immune evasion might contribute to the limited effectiveness of current immunotherapies for OC. Furthermore, studies have reported that GA, acting as an AhR antagonist, can competitively bind to its ligands, thereby inhibiting AhR activation and preventing tumor progression [40,41]. In light of this, this study aimed to investigate the anti-tumor mechanism of GA by regulating Stat3/IDO1/AhR signaling axis. First, cell viability experiments demonstrated that GA significantly suppressed the proliferation of OC cells (Fig. S4). Western blotting results showed that GA markedly inhibited the phosphorylation levels of Jak1 and Stat3, and simultaneously reduced the expression of IDO1 and AhR proteins in a dose-dependent manner (Fig. 1E and F). Collectively, these results indicate that GA directly binds to Stat3 and accelerates its degradation, thereby exerting its anti-tumor effects by inhibiting Stat3/IDO1/AhR signaling pathway.

Furthermore, it has been reported that GA's targeted inhibition of PD-L1 enhances the activity of T cells, thereby exerting potent anti-tumor effects [24]. To investigate the correlation between the anti-tumor activity and immune activation of GA, the impact of GA on the induction of immunogenic cell death (ICD) in tumor cells was evaluated. The occurrence of ICD leads to the release of damage-associated molecular patterns (DAMPs) such as high mobility group box 1 protein (HMGB1), calreticulin (CRT), and ATP, which trigger immune responses against tumors [33]. CRT on cell surfaces binds to DC receptors, HMGB1 released from nuclei binds TLR4, and ATP secreted *via* lysosomal rupture recruits DCs, collectively activating cytotoxic T lymphocytes (CTLs) to enhance the immune response and tumor cell eradication [42,43]. Simultaneously, studies have reported that Stat3 antagonists can effectively degrade Stat3 protein in the tumor microenvironment, promote the maturation and function of DCs, thereby enhancing anti-tumor immune responses [15]. The immunofluorescence results showed that GA induced ICD in tumor cells, characterized by increasing the exposure of CRT on the cell membrane, migrating of HMGB1 from nuclei, elevating extracellular ATP levels (Fig. 1G–I). Further investigation was conducted using an *in vitro* model in which spleen lymphocytes from healthy C57 mice were co-cultured with ID8 cells at a ratio of 1:10. The results revealed that GA significantly promoted T cell proliferation and increased the proportion of CD8^+^ T cells (Fig. 1J and K), indicating GA stimulates the immune response activation. Overall, these findings suggest that GA not only kills tumor cells by inhibiting the Stat3/IDO1/AhR signaling axis, but also induces ICD to activate the immune response, making GA a potential candidate drug for ovarian cancer.

### 3.2 Construction and characterization of GA-NPs@DCV

**Fig. 2:**
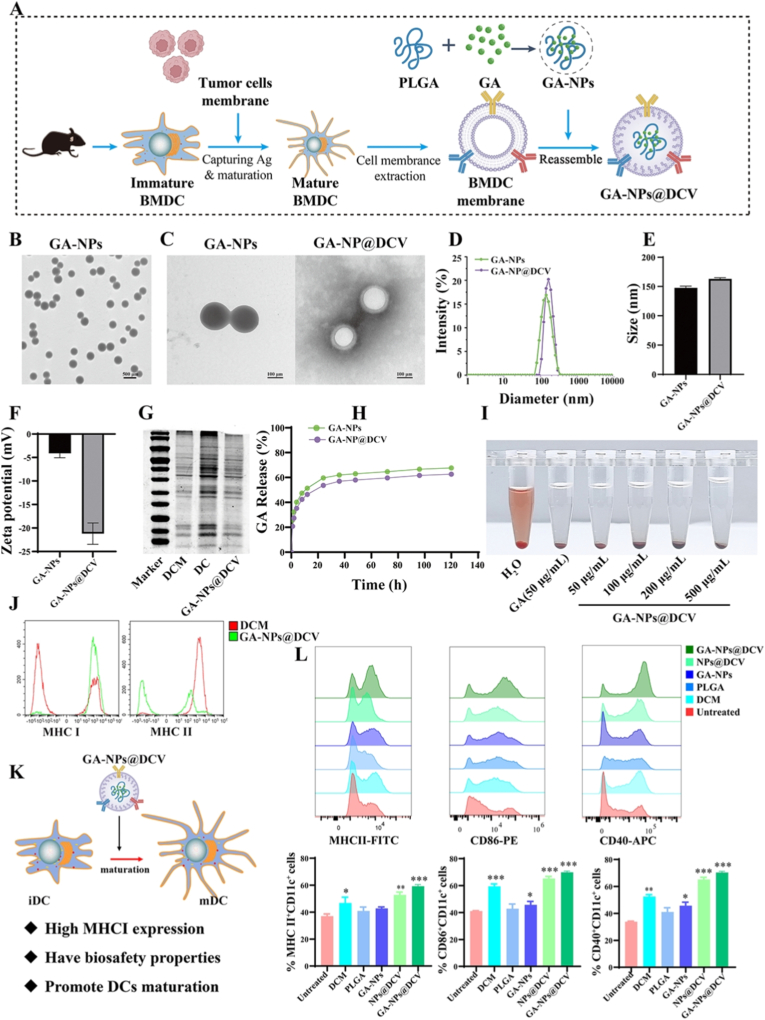
Construction and characterization of GA-NPs@DCV. A) A schematic illustration of the construction of GA-NPs@DCV, which were fabricated by coating GA loaded PLGA-NPs with DC membrane from the maturation BMDCs pulsed with ID8 cells membrane. B, C) TEM images of GA-NPs and GA-NPs@DCV. D, E, F) Particle size distribution, ζ-potential and particle size of the nanoparticles GA-NPs and GA-NPs@DCV. G) Total protein profiles of DCs, DCM, GA-NPs@DCV on SDS- PAGE. H) The release of GA *in vitro* was detected by HPLC. I) Hemolysis test was used to detect the biological safety of GA-NPs@DCV. J) MHC I and MHC II contents of DCM and GA-NPs@DCV detected by flow cytometry. K, L) Schematic diagram of GA-NPs@DCV promoting DC and flow cytometry results. Data are presented as mean ± SD and *p*-values <0.05 were considered significant (∗*p* < 0.05, ∗∗*p* < 0.01, ∗∗∗*p* < 0.001).

Although GA presents potent anti-tumor activity, the unsatisfactory solubility and bioavailability strictly restrict its application [44]. Similarly, DC-based vaccines have shown promise in immunotherapy for enhancing antigen-specific immune responses against tumors. However, its clinical efficacy is constrained by the limited presentation of tumor-associated antigens (TAAs) *via* MHC I molecules and a low efficiency of lymph node homing [5,45]. Therefore, to enhance targeting and improve the therapeutic efficacy of GA and DC vaccines, this study employed a composite emulsion method to encapsulate GA within PLGA nanoparticles, and further utilized DC membrane-coated nanoparticle technology (Fig. 2A). As shown in Fig. 2B, GA was loaded into PLGA nanoparticles (GA-NPs) during synthesis, with transmission electron microscopy (TEM) images revealing the uniform spherical structure of the GA-NPs.

To enhance the targeting of DC vaccines, bone marrow-derived dendritic cells (BMDCs) were pulsed with the ID8 cell membrane, effectively improving the uptake of TAAs and promoting DC maturation, characterized by high expression of MHC II, CD40, and CD86 (Fig. S5). The mature BMDC membrane (DCM) was isolated and subsequently extruded with GA-NPs to fabricate a GA-loaded nano-DC vaccine (GA-NPs@DCV) (Fig. 2A). TEM images showed that GA-NPs were completely enveloped within DCM, resulting in nano-DC vaccines with a core-shell structure (Fig. 2C). The diameter was approximately 144.8-164.8 nm (Fig. 2D). Notably, the average diameter of less than 200 nm may facilitate their free transport to lymph nodes [46]. Dynamic light scattering indicated an increase of approximately 20 nm in the diameter of GA-NPs@DCV, consistent with the thickness of the bilayer cell membrane (Fig. 2E). The **ζ-**potential of GA-NPs@DCV was decreased from −4.1 mV to −21.5 mV (Fig. 2F), which enhanced its stability and prolonged its blood circulation time. As described in Fig. 2H, the immunoblotting showed that most proteins were retained in DCM, DC, and GA-NPs@DCV during the isolation of the DC membranes. To determine the GA loading capacity, high-performance liquid chromatography (HPLC) was employed. The results revealed that the GA loading capacity of GA-NPs@DCV was 33.6%. Furthermore, in PBS at 37 °C, both GA-NPs and GA-NPs@DCV exhibited a burst release of GA within the first 12 h, showing similar cumulative release over 7 days (Fig. 2G), indicating that GA-NPs and GA-NPs@DCV exhibited excellent biological stability.

Cells viability measured by MTT assays revealed that GA-NPs@DCV exhibited no significant cytotoxicity toward normal cells (RAW.267.4, 293T cells), while significantly suppressing the proliferation of ID8 tumor cells and inducing tumor cell death (Fig. S6). Further validation of cellular uptake demonstrated that DID-labeled GA-NPs@DCV was indeed internalized by tumor cells. Combined with the significant *in vitro* cytotoxicity demonstrated earlier, these results strongly support our hypothesis that GA-NPs@DCV can exert a direct tumoricidal effect. As demonstrated in Fig. 2I, a hemolysis test further verified the biosafety of GA-NPs@DCV. PLGA nanoparticles have been reported to possess the capability of both antigen delivery and acting as adjuvants, thereby enhancing the abscopal effect and cancer immunotherapy through improved T cell responses and the activation of antigen-presenting cells [28,47]. As shown in Fig. 2L, treatment with empty PLGA NPs induced a slight, but not statistically significant, increase in the expression of co-stimulatory molecules on DCs compared to the untreated group. Further evaluation of the proportion of mature BMDCs (CD11c^+^ MHC II^+^, CD11c^+^CD40^+^ and CD11c^+^CD86^+^) were also increased administration with DCM. The synergy between the antigen-presenting DCM and the PLGA nanoparticle core drives this potent immune activation, highlighting the advantage of this strategy over using nanoparticles as a passive delivery vehicle. Moreover, the results presented in Fig. 2K and L demonstrate that GA-NPs@DCV significantly upregulates MHC-I expression, and promotes the maturation of DCs through co-stimulatory factors and antigen cross-presentation. In summary, the biomimetic GA-NPs@DCV with high expression of MHC I, excellent biosafety and biostability was successfully constructed, laying a solid foundation for subsequent experiments. Notably, the average diameter of GA-NPs@DCV was less than 200 nm (Fig. 2D), a size range reported to be optimal for efficient drainage and transport to lymph nodes [46]. This facilitates DC maturation and triggers an anti-tumor immune response, further supporting the feasibility of this therapeutic strategy.

### 3.3 Antigen-specific CTL responses induced by GA-NPs@DCV

**Fig. 3:**
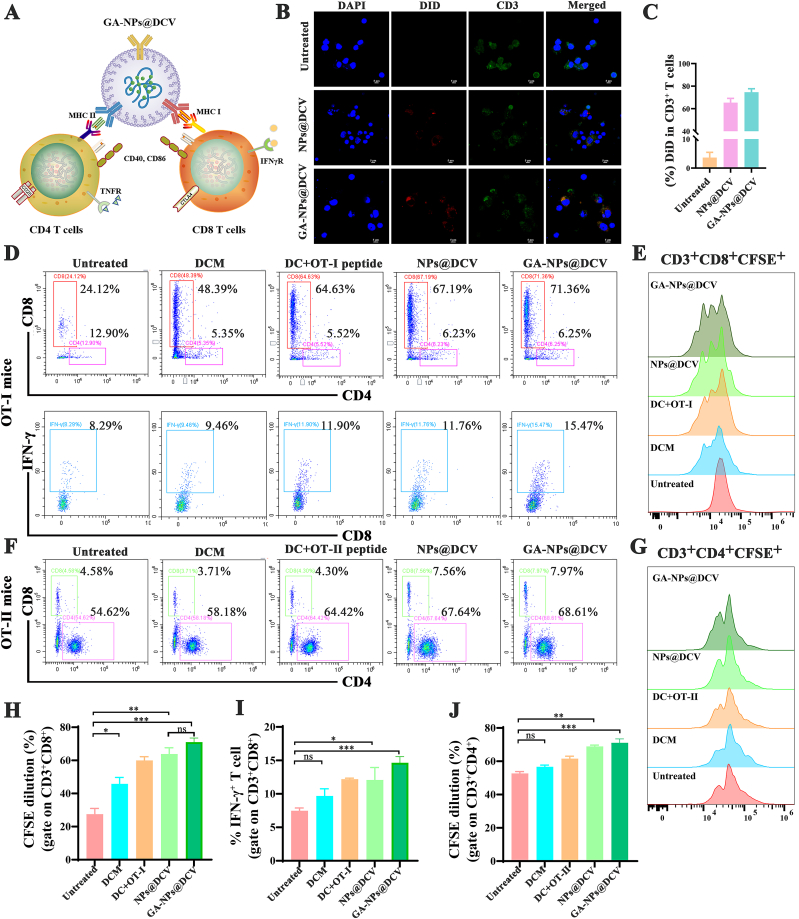
GA-NPs@DCV induces antigen-specific CTL responses *in vitro*. A) Schematic diagram illustrating the mechanism of GA-NPs@DCV activate immune responses. B) CLSM images of splenic lymphocytes incubated with DID-labeled NPs@DCV and GA-NPs@DCV at 4 °C for 1 h. Blue represents the cell nucleus, red represents nanoparticles, and green represents CD3^+^ T cells. C) Relative DID intensity within the CD3^+^ T cells cultured with different nanoparticles. D, E, H) Flow cytometric analysis, histograms, and percentage of CD3^+^CD8^+^CFSE^+^ T cells in CFSE-labeled spleen lymphocytes from OT-I transgenic mice incubation with various formulations for 72 h. D, I) Flow cytometric analysis and percentage of CD3^+^CD8^+^ IFN-γ^+^ T cells in CFSE-labeled spleen lymphocytes from OT-I transgenic mice incubation with various formulations for 72 h. F, G, J) Flow cytometric analysis, histograms, and percentage of CD3^+^CD4^+^CFSE^+^ T cells in CFSE-labeled spleen lymphocytes from OT-Ⅱ transgenic mice incubation with various formulations for 72 h. OT-I peptide is T cell receptor-specific antigenic peptide OVA 257-264 and OT-II peptide is T cell receptor-specific antigenic peptide OVA323-339. Data are presented as mean ± SD and *p*-values <0.05 were considered significant (∗*p* < 0.05, ∗∗*p* < 0.01, ∗∗∗*p* < 0.001).

To investigate the interaction between GA-NPs@DCV and T cells in activating the immune response (Fig. 3A), we performed a quantitative flow cytometric analysis. Splenic lymphocytes were incubated with DID-labeled NPs@DCV or GA-NPs@DCV. As revealed in Fig. 3B, a significantly higher DiD fluorescence signal was detected in CD3^+^ T cells incubated with our nano DC vaccines compared to the untreated group. This finding was further confirmed by flow cytometric analysis (Fig. 3C). This result provides robust evidence that the nano-DC vaccines can effectively and specifically bind to T lymphocytes, establishing a crucial foundation for the subsequent antigen-specific T cell activation.

Based on the above results, the T cell receptor-specific antigenic peptides OVA257-264 and OVA323-339 (OT-I and OT-II peptides, respectively) were used to prepare GA-NPs@DCV. This aimed to further elucidate the specific activation of CTLs by GA-NPs@DCV and the subsequent enhancement of the immune response. Antigen-specific activation of T cells by DC vaccines is a crucial step in initiating the immune response to elicit their anti-tumor effects [48]. Next, splenic lymphocytes from OT-I/OT-Ⅱ transgenic mice were labeled with CFSE and incubated with corresponding antigen-carrying GA-NPs@DCV to assess the proliferation of CD4^+^ and CD8^+^ T cells. As depicted in Fig. 3D–G, flow cytometric analysis revealed that treatment with DC + OVA257-264 or OVA323-339, DCM, NPs@DCV, and GA-NPs@DCV significantly enhanced the proliferation of both CD8^+^ T cells (Fig. 3D–H) and CD4^+^ T cells (Fig. 3F–J). A particularly marked increase was observed in CD8^+^ T cell proliferation, likely due to the high expression of MHC I on GA-NPs@DCV. Additionally, compared to the untreated group, NPs@DCV and GA-NPs@DCV strongly promoted the differentiation of CD8^+^ IFN-γ^+^ T cells, indicating the activation of CTL (Fig. 3D–I). GA-NPs@DCV employs a dual-pathway mechanism, combining direct T-cell activation *via* pre-processed pMHC complexes (Fig. 3A), and act as an adjuvant by enhancing the maturation of DCs through cross-presentation of antigens (Fig. 2L). This synergistic strategy generates a rapid, specific, and durable anti-tumor immune response, offering a key advantage over traditional DC vaccines. Taken together, these findings demonstrate that GA-NPs@DCV effectively triggered antigen-specific responses, thereby contributing to its anti-tumor effects. This provides a robust experimental basis for subsequent studies on enhancing antigen presentation and targeted tumor therapy using GA-NPs@DCV.

### 3.4 Antitumor immune response stimulated by GA-NPs@DCV *via* Stat3/IDO1/AhR signaling axis *in vitro*

**Fig. 4:**
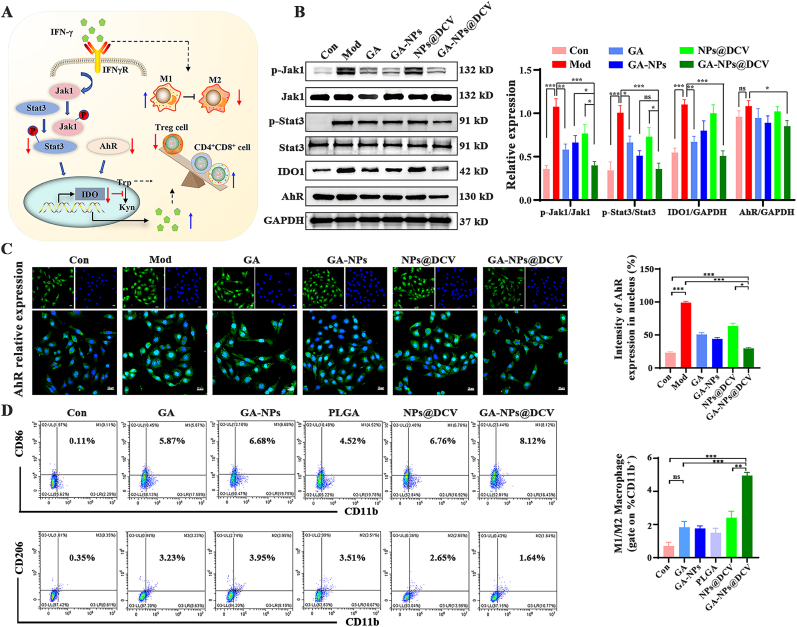
GA-NPs@DCV inhibits Stat3/IDO1/AhR signaling axis to stimulate antitumor immune response *in vitro*. A) Schematic illustration of the suppression tumor immune escape *via* inhibiting Stat3/IDO1/AhR axis by GA-NPs@DCV. B) Representative western blots analysis of Jak1, p-Jak1, Stat3, p-Stat3, IDO1, and AhR after treatment with the different formations in IFN-γ-induced ID8 cells. C) Immunofluorescence staining of AhR in ID8 cells after different forms of administration. Blue indicated the nucleus and green indicated the expression of AHR protein, scale bar = 20 μm. D) Flow cytometric analysis and percentage of M1-type (CD11b^+^CD86^+^ cells) and M2-type (CD11b^+^CD206^+^ cells) macrophages after incubation with various formulations for 72 h. Data are presented as mean ± SD and *p*-values <0.05 were considered significant (∗*p* < 0.05, ∗∗*p* < 0.01, ∗∗∗*p* < 0.001).

Inspired by the ability of GA-NPs@DCV to activate antigen-specific activate T cells, we subsequently investigated the mechanisms by which GA-NPs@DCV activated immune cells and inhibited tumor immune evasion *in vitro*. The Stat3/IDO1/AhR axis plays a crucial role in tumor immune evasion by modulating the tumor microenvironment. IDO1 expression, often induced by IFN-γ *via* the Jak-Stat signaling pathway, promotes the recruitment of immunosuppressive cells and depletes tryptophan, inhibiting T cell responses [18,39,49]. Furthermore, AhR activation, downstream of IDO1, contributes to increased tumor aggressiveness and immune checkpoints [16,50,51]. Based on our previous findings, GA directly bound to Stat3, exerting anti-tumor effects through the inhibition of the Stat3/IDO1/AhR pathway (Fig. 1D). Therefore, the suppression of tumor immune escape *via* inhibiting Stat3/IDO1/AhR axis by GA-NPs@DCV was investigated (Fig. 4A). In IFN-γ-induced ID8 cells, GA-NPs@DCV administration significantly inhibited the phosphorylation levels of Jak1 and Stat3, markedly reducing the expression levels of IDO1 and AhR compared to the control group (Fig. 4B). Moreover, we also detected the expression levels of the key tumor immune evasion hotspot molecule AhR by immunofluorescence analysis. GA-NPs@DCV markedly inhibited the nuclear translocation of AhR, resulting in decreased its expression levels and limiting its activation, which was consistent with the Western blotting results (Fig. 4C). These findings indicate that GA-NPs@DCV exhibit anti-tumor activity through blocking tumor immune evasion by inhibiting Stat3/IDO1/AhR signaling axis.

Tumor immune microenvironment (TIME) is composed of effector T cells, regulatory T cells (Tregs), macrophages and DCs. However, tumor immune escape *via* antigen restriction, immune inhibition, and metabolic reprogramming within the TIME limits immunotherapy efficacy [52]. Suppressing immunosuppressive immune cells while activating anti-tumor immune cells represents an ideal and effective strategy for cancer immunotherapy [53,54]. To assess the activation of immune cells, splenic lymphocytes were treated with different formulations (GA, GA-NPs, PLGA, NP@DCV, and GA-NPs@DCV) for 3 days, followed by flow cytometry analysis. As shown in Fig. S7, compared to the untreated group, GA-NPs@DCV significantly promoted T cell proliferation, increased the proportion of CD8^+^ T cells and CD4^+^ T cells, reduced Tregs differentiation. Furthermore, GA-NPs@DCV markedly facilitated the M1-type polarization of macrophage (CD11b^+^CD86^+^ cells) while inhibiting M2-type polarization (CD11b^+^CD206^+^ cells) (Fig. 4D). Collectively, these findings demonstrate that GA-NPs@DCV not only activates immune cells to enhance the immune response but also inhibits tumor evasion by suppressing the Stat3/IDO1/AhR signaling axis, thereby exerting anti-tumor activity.

### 3.5 The biosafety of GA-NPs@DCV and accumulation in lymph nodes and tumor

The acute toxicity study confirmed the excellent biocompatibility and high degree of biosafety of the GA-NPs@DCV platform. Throughout the 14-day observation period at doses up to 100 mg/kg, no mortality or significant signs of toxicity were observed (Fig. S8). This was substantiated by stable body weights (Fig. S8A), normal serum biomarkers, such as ALT, AST, BUN, Ser, and CK-MB (Fig. S8B–F) for liver, kidney, and cardiac function, and the absence of pathological damage in major organs upon histopathological examination (Fig. S8J). A particularly encouraging finding was the significant increase in immune cell populations within the spleen, which not only confirms the vaccine's safety but also demonstrates its intended function as a potent immunostimulatory agent. This favorable safety profile, combined with clear evidence of immune activation, provides compelling support for the advancement of GA-NPs@DCV toward further preclinical development and future clinical translation.

**Fig. 5:**
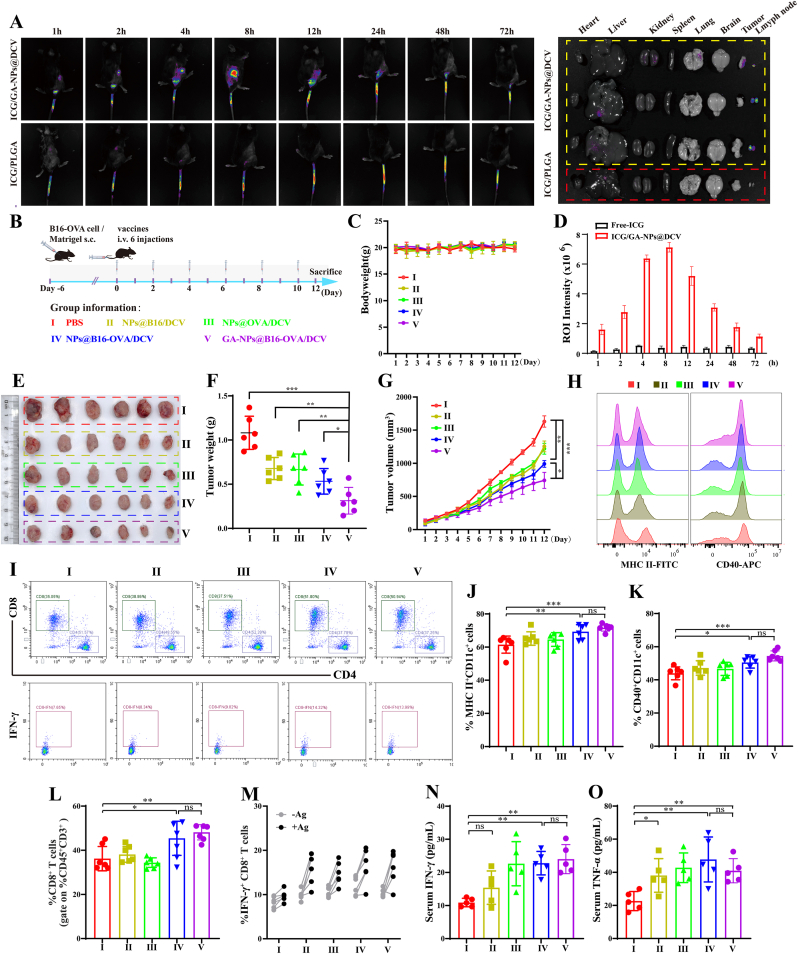
Specific anti-tumor immune stimulation ability and therapeutic efficacy of a carrying different antigen NPs@DCV in melanoma-bearing mice. A) The fluorescence of GA-NPs@DCV labeled with ICG through the IVIS Lumina XRMS Series Imaging System. B) Schematic illustration for establishing melanoma-bearing mice and their treatment (n ≥ 6). C) Body weight changes of tumor-bearing mice during treatment. D, E) Photographs of lymph nodes and tumor tissue at the end of the experiment. F, G) Tumor volume changes during treatment and tumor weight at the end of the experiment. H-K) Representative flow cytometry histograms and percentage of CD40^+^ and MHC II^+^ in spleen each group of melanoma-bearing mice after administration with various formations. L) Flow cytometric analysis and percentage of CD3^+^CD8^+^ T cells in spleen each group of melanoma-bearing mice after administration with various formations. M) The antigen-specific response tests were conducted that spleen cells from vaccinated melanoma subcutaneous tumor mice, upon re-exposure to the corresponding antigen. Flow cytometric analysis and percentage of CD8^+^IFN-γ^+^ T cells, indicating the activation of an immune response to effectively prevent tumor recurrence. N, O) The TNF-α, and IFN-γ secreted in the serum analyzed by enzyme-linked immunosorbent assay (ELISA). Data are presented as mean ± SD and *p*-values <0.05 were considered significant (∗*p* < 0.05, ∗∗*p* < 0.01, ∗∗∗*p* < 0.001).

Considering that GA-NPs@DCV possessed mature DC membranes carrying tumor antigens, which could facilitate DC maturation and activate T cells immune responses specifically *in vitro*, we further examined the *in vivo* distribution of GA-NPs@DCV. Migration of the vaccine to lymph nodes, tumor microenvironment, and subsequent retention therein, largely determines the effectiveness of vaccine-induced activation of immune responses [55]. Indocyanine green (ICG)-labeled GA-NPs@DCV was injected into the tail vein of ID8 tumor-bearing mice, with the same dose of ICG-labeled PLGA-NPs serving as a control. Initially, the optimal dosage of ICG-labeled GA-NPs@DCV administered was explored. *In vivo* living imaging results revealed that a DCM: GA-NPs at the ratio of 1:1 resulted in the best targeting to lymph nodes and tumor tissues (Fig. S1). As shown in Fig. 5A, the accumulation of ICG-labeled GA-NPs@DCV in the inguinal lymph nodes and tumor tissues increased significantly from 1 h to 8 h post-injection. In contrast, ICG-labeled PLGA-NPs did not exhibit marked accumulation, with any observed faint fluorescence being primarily attributed to hepatic and renal metabolism of ICG and PLGA. Between 12 and 72 h, the fluorescence of ICG-labeled GA-NPs@DCV intervention group gradually diminished. At 72 h, the major tissues were collected, and the accumulation of ICG-labeled GA-NPs@DCV was determined using a small animal living imaging system. Excitingly, compared to the control group, ICG-labeled GA-NPs@DCV exhibited marked migration and accumulation in inguinal lymph nodes and tumor tissues (Fig. 5A–D). These findings suggested that GA-NPs@DCV could effectively accumulate in the lymph nodes and tumor tissues due to their unique physicochemical characteristics (average diameter <200 nm, immunoactivity, etc.). This homing ability of GA-NPs@DCV ensures its potential for subsequent *in vivo* immune activation and antitumor activity.

### 3.6 Specific anti-tumor immune response induced by NPs@DCV in melanoma-bearing mice

In light of the excellent immune activation and targeted homing capabilities of GA-NPs@DCV, it draws our attention to its anti-tumor effect *via* initiating specific immune response. As shown in Fig. 5B, we immunized melanoma-bearing mice with NPs@DCV carrying different antigens (B16, B16-OVA, and OVA) as well as GA-NPs@B16-OVA/DCV for a total of six administrations. No significant differences in body weight or tissue indices of major organs were observed among groups of mice (Fig. 5C). Compared with Group I, Groups II-V significantly inhibited tumor growth in the subcutaneous melanoma mouse model, accompanied by a notable enlargement of the inguinal lymph nodes, with the most prominent in Group V (Fig. S9A and Fig. 5E–G). These results suggest that Group V possessed antitumor activity without significant biotoxicity, thus demonstrating its biosafety.

Furthermore, significant proliferation of anti-tumor immune cells was observed in the lymph nodes and spleen tissues of mice in Groups IV and V. This included increases in CD3^+^ T cells, CD4^+^ T cells, CD8^+^ T cells, DCs, and M1-type macrophages, while simultaneously decreases immunosuppressive cells that promote tumor growth, such as M2-type macrophages (Fig. 4H–L and Fig. S9B–F). The populations of CD8^+^ T cells and M1-type polarization (CD11b^+^CD86^+^ cells) were significant increased, while decreased M2-type polarization (CD11b^+^CD206^+^ cells) within the tumor microenvironment (Fig. S9G–I). The elevation in the proportion of CD8^+^ IFN-γ^+^ T cells enhances the ability to recognize and kill tumor cells, inhibits tumor cell growth, activates other immune cells, and promotes the formation of immune memory, thereby improving the efficacy of antitumor immune therapy [56]. Therefore, antigen-specific response tests were conducted. The results showed that spleen lymphocytes from vaccinated melanoma subcutaneous tumor mice, upon re-exposure to the corresponding antigen, rapidly activated an immune response, indicating an effective prevention of tumor recurrence. Excitingly, Groups IV and V displayed the most significant effect in elevating CD8^+^IFN-γ^+^ T cells, thereby generating a long-term protective anti-tumor immune response (Fig. 5L and M). Additionally, Groups IV was shown to enhance the levels of immune cytokines IFN-γ and TNF-α in serum (Fig. 5N and O). These findings indicate that both Groups IV and V not only inhibit tumor growth and activate systemic immune responses but also possess the capacity to effectively prevent tumor recurrence. This suggests that nano-DC vaccines hold promise for providing novel therapeutic strategies in tumor immunotherapy.

### 3.7 GA-NPs@DCV reprogram the tumor microenvironment to boost immunotherapy in ovarian cancer-bearing mice

**Fig. 6:**
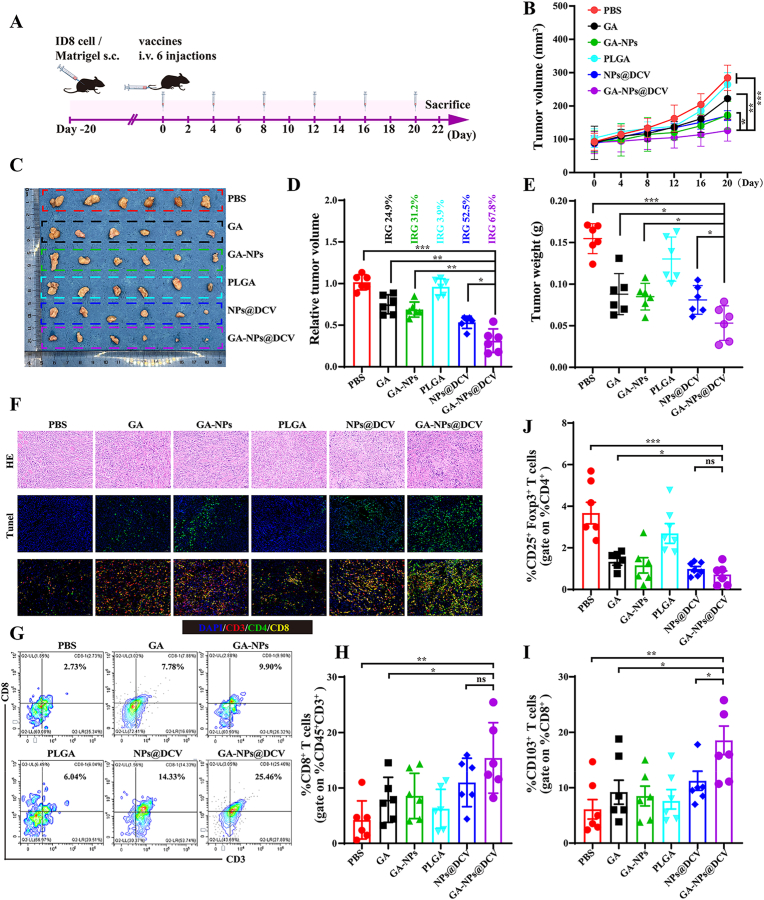
GA-NPs@DCV exerts anti-tumor effects by boosting immune responses for long-term immune memory in ovarian cancer bearing mice. A) Schematic illustration of s.c. ID8 and treatment with different formations (n ≥ 6). B) Tumor volume changes during experiment strategies. C) The image of tumor tissue at the end of the experiment. D, E) Relative tumor volume changes and tumor weight at the end of the experiment. F) HE staining, Tunel stanning, and immunofluorescence staning of CD3^+^, CD4^+^, CD8^+^ T cells of tumor tissue. G-J) Flow cytometric analysis and percentage of CD3^+^CD8^+^, Treg (CD25^+^Foxp3^+^), T_RM_ (CD8^+^CD103^+^) T cells in tumor tissue each group of ovarian cancer bearing mice after administration with various formations. Data are presented as mean ± SD and *p*-values <0.05 were considered significant (∗*p* < 0.05, ∗∗*p* < 0.01, ∗∗∗*p* < 0.001).

The efficacy of GA-NPs@DCV in specifically activating T cells prompted us to investigate whether the antitumor immune response it induces can inhibit tumor growth in ovarian cancer-bearing mice, and to explore the underlying mechanisms. An ovarian cancer model was established by subcutaneously injecting ID8 cells into the right flank of female C57BL/6 mice. When the tumor volume reached ≥100 mm^3^, the mice were administered PBS, GA, GA-NPs, PLGA, NPs@DCV, or GA-NPs@DCV *via* tail vein injection every four days, mimicking a clinically relevant model of ovarian cancer therapeutic efficacy rather than prophylaxis (Fig. 6A). Compared to the PBS group, PLGA did not exhibit significant tumor growth inhibition. In contrast, GA, GA-NPs, NPs@DCV, and GA-NPs@DCV were found to delay tumor growth following inoculation, with GA-NPs@DCV demonstrating the greatest therapeutic effect at the end of monitoring (Fig. 6B–E). To elucidate the mechanism underlying the enhanced antitumor effects of GA-NPs@DCV, we collected tumor tissues for H&E staining, terminal deoxynucleotidyl transferase dUTP nick end labeling (Tunel) staining, and immunofluorescence analysis of CD3, CD4, and CD8 T cells. The results indicate that mice vaccinated with GA-NPs@DCV exhibited a significant reduction in tumor cells and a marked increase in the number of CD8^+^ T lymphocytes (CTLs) compared to PBS groups (Fig. 6F). The increased infiltration of CTLs within the tumors likely contributed to significant tumor cell apoptosis, as demonstrated by Tunel staining (Fig. 6F). These findings are consistent with the predictive value of tumor-infiltrating CD8^+^ T cells for survival outcomes in human ovarian cancer patients [57]. Consistently, flow cytometry also confirmed that NPs@DCV and GA-NPs@DCV significantly augmented the proliferation of antitumor immune cells in the tumor tissue, including CD3^+^CD8^+^ T cells (Fig. 6G and H), while suppressing the immunosuppressive Tregs (CD25^+^Foxp3^+^ cells) that promote tumor growth (Fig. 6J). Moreover, tissue-resident memory T cells (T_RM_), especially CD8^+^CD103^+^T_RM_, play a critical role in effective tumor cell clearance through rapid recognition and potent cytotoxic activity, as well as by assisting other immune cells, thus sustaining long-term immune memory and preventing tumor recurrence [58,59]. Targeting the activation of T_RM_ or their infiltration into the tumor microenvironment may improve the prognosis of ovarian cancer patients and become an important direction for future immunotherapy. Flow cytometric analysis of T_RM_ in the tumor tissue indicated that the GA-NPs@DCV treatment significantly increased the number of CD8^+^CD103^+^ cells (Fig. 6I), suggesting a potential for maintaining long-term immune memory and preventing tumor relapse.

**Fig. 7:**
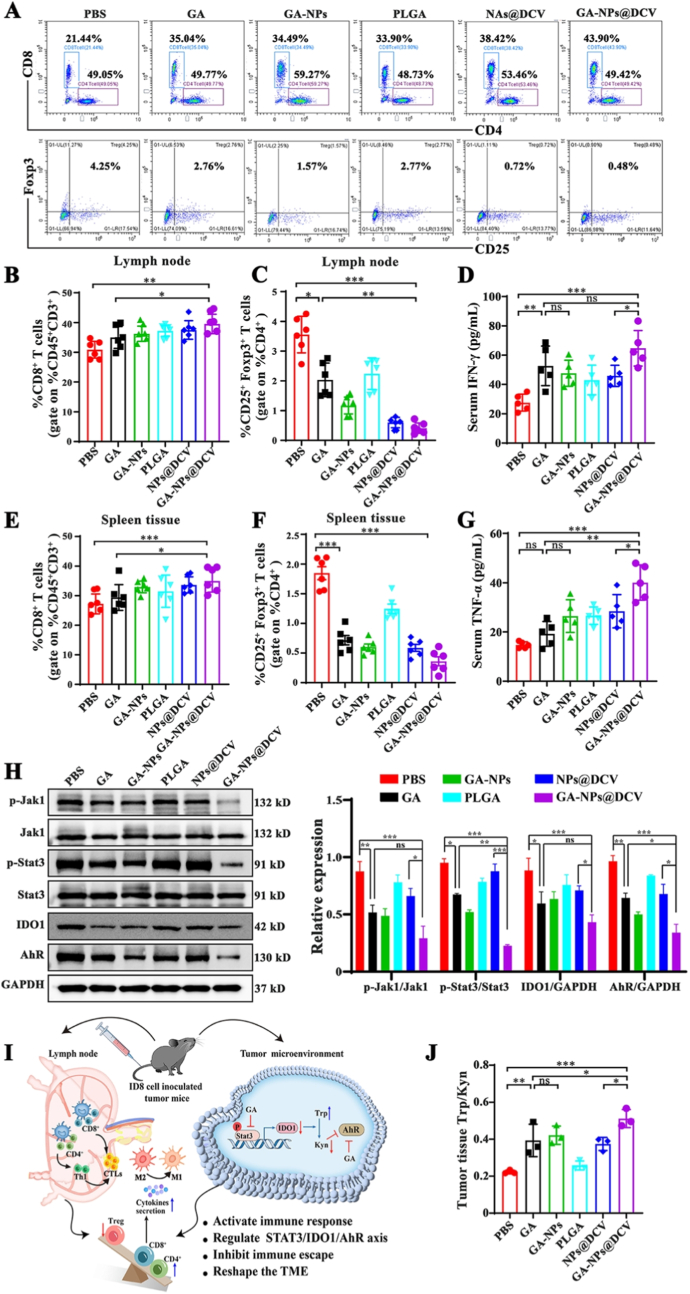
GA-NPs@DCV reprograms the tumor microenvironment to boost immunotherapy in ovarian cancer mice. A-C) Flow cytometric analysis and percentage of CD3^+^CD8^+^, Treg (CD25^+^Foxp3^+^) T cells in lymph nodes each group of ovarian cancer bearing mice after administration with various formations. E, F) The percentage of CD3^+^CD8^+^, Treg (CD25^+^Foxp3^+^) T cells in spleen each group. D, F) The TNF-α, and IFN-γ secreted in the serum analyzed by enzyme-linked immunosorbent assay (ELISA). H) Representative western blots analysis of Jak1, p-Jak1, Stat3, p-Stat3, IDO1, and AhR in tumor tissue after treatment with the different formations in ovarian cancer bearing mice. I) Mechanism diagram illustrating that GA-NPs@DCV exerts prevent anti-tumor effects by inhibiting tumor escape through the Stat3/IDO1/AhR signaling axis, reshaping the immune microenvironment by modulating tryptophan metabolism, activating systemic and local immune responses. J) The concentration levels of tryptophan (Trp) and kynurenine (Kyn) in tumor tissues examined by HPLC and presented the ratio of Trp to Kyn. Data are presented as mean ± SD and *p*-values <0.05 were considered significant (∗*p* < 0.05, ∗∗*p* < 0.01, ∗∗∗*p* < 0.001).

Furthermore, the up-regulation of CD3^+^CD8^+^ effector T cells and CD3^+^CD4^+^ T cells in the lymph nodes and spleens of mice treated with GA-NPs@DCV was the most significant compared with the other groups (Fig. 7A, B, D). In contrast, the proportion of CD4^+^CD25^+^Foxp3^+^ regulatory T cells in the lymph nodes and spleens of mice immunized with GA-NPs@DCV was lower than that in other groups (Fig. 7A–C, E). Administration of GA-NPs@DCV also induced the proliferation of M1-type macrophages, while inhibiting M2-type macrophages in the spleens of each group, indicating the activation of enhanced anti-tumor immune responses (Fig. S10). Moreover, NPs@DCV and GA-NPs@DCV also markedly promoted the increase of immune cytokines IFN-γ and TNF-α in the serum, suggesting the activation of a systemic immune response (Fig. 7F and G). During the experiment, the *in vivo* biosafety of GA-NPs@DCV was evaluated. All the treatment mice maintained stable body weight throughout the entire experiment (Fig. S11A). Consistently, serum biochemical analysis of indicators for liver (ALT, AST), kidney (BUN, Cr), and cardiac (CK-MB) function showed that levels were within normal reference ranges in all groups at the conclusion of the study (Fig. S11B–F). Furthermore, H&E staining of major organs also revealed no obvious pathological changes, collectively confirming the biosafety of GA-NPs@DCV (Fig. S11J). The aforementioned results indicate that GA-NPs@DCV demonstrates a favorable safety profile, activates systemic as well as local immune responses, thereby exerting an anti-tumor effect.

*In vitro* studies demonstrated that GA and GA-NPs@DCV mitigated tumor escape mechanisms by inhibiting the Stat3/IDO1/AhR signaling axis, thereby enhancing immune responses. Herein, examination of key proteins related to Stat3/IDO1/AhR in tumor tissues showed that GA-NPs@DCV treatment significantly inhibited the phosphorylation levels of Jak1 and Stat3, and significantly reduced the expression levels of IDO and AhR (Fig. 7I). Conversely, the NPs@DCV group exhibited subtle changes, highlighting the synergistic antitumor mechanism of GA in conjunction with NPs@DCV. Additionally, the measurement of tryptophan (Trp) and kynurenine (Kyn) levels in the tumor tissues indicated that GA-NPs@DCV administration significantly increased Trp concentration, while decreased the concentration of Kyn, further confirming the suppression of IDO1 activity (Fig. 7H–J).

Trp is an essential amino acid required for T cell activation, and its elevation suggests a potential to activate both systemic and local immune responses, thereby exerting antitumor effects [49]. As shown in Fig. 7J, the Trp/Kyn ratio in tumor tissue homogenates was significantly increased following treatment with GA and GA-NPs@DCV. As evidenced by the altered Trp/Kyn ratio and the regulation of the Stat3/IDO1/AhR axis, GA-NPs@DCV effectively alleviated immunosuppression and remodeled the immune microenvironment through tryptophan metabolism. In summary, these results confirmed that GA-NPs@DCV inhibits tumor escape *via* the Stat3/IDO1/AhR signaling axis, reshapes the immune microenvironment by regulating tryptophan metabolism, activates both systemic and local immune responses, and maintains long-term immune memory, thereby exhibiting the potential for anti-tumor effects and preventing tumor recurrence. While the subcutaneous ID8 model was chosen for its high reproducibility in a preliminary proof-of-concept study, it does not fully recapitulate the intraperitoneal dissemination of human ovarian cancer. To address this, follow-up studies in a more clinically relevant in situ ovarian cancer mouse model are already showing promising results, representing a critical next step in demonstrating the nano-vaccine's translational potential. This approach offers a novel strategy for targeted ovarian cancer immunotherapy.

## 4 Conclusions

This study focuses on ovarian cancer, a highly recurrent and low-survival-rate gynecological malignancy. Addressing the limitations of existing immunotherapies (such as DC vaccines), we successfully developed a novel biomimetic nano-DC vaccine, GA-NPs@DCV. This vaccine integrates the tumor ICD effect induced by GA and targeting capability mediated *via* the mature DC membrane pulsed with tumor cell membranes. Compared to traditional DC vaccines, GA-NPs@DCV enhances antigen presentation both *in vitro* and *in vivo*, effectively targets lymph nodes and the tumor microenvironment to elicit a more robust T cell response. This enhancement may be attributed to the nanoscale effect, which helps overcome temporal and spatial barriers in antigen presentation. Further validation experiments confirmed that GA-NPs@DCV exhibits potent anti-tumor effects and establishes long-term immune memory by inhibiting immune escape through the Stat3/IDO1/AhR axis, reshaping the tumor immune microenvironment *via* tryptophan metabolism modulation, and inducing tissue-resident memory T cells (T_RM_, CD8^+^CD103^+^). Collectively, our findings establish GA-NPs@DCV as a versatile, personalized nano DC vaccine platform with clear translational potential for ovarian-cancer immunotherapy. Future work will focus on validating its efficacy in heterogeneous patient-derived models, optimizing large-scale manufacturing, and exploring combination regimens with checkpoint blockade to further amplify clinical benefit.

## CRediT authorship contribution statement

**Nuerbiye Aobulikasimu:** Conceptualization, Data curation, Funding acquisition, Writing – original draft, Writing – review & editing. **Lele Fang:** Investigation, Methodology, Software, Writing – original draft. **Aidiresi Maimaitiyiming:** Conceptualization, Formal analysis, Validation, Writing – review & editing. **Dilimureti Kasimu:** Methodology, Software, Writing – original draft. **Adila Aipire:** Data curation, Funding acquisition, Investigation. **Weilan Wang:** Investigation, Methodology, Validation. **Zhongxiong Fan:** Conceptualization, Methodology, Validation, Visualization. **Jinyao Li:** Conceptualization, Funding acquisition, Project administration.

## Declaration of competing interest

The authors declare that they have no known competing financial interests or personal relationships that could have appeared to influence the work reported in this paper.

## Data Availability

Data will be made available on request.
