# Supplementary material for: Galangin-loaded biomimetic dendritic cells membrane nanovaccine reprograms the ovarian cancer microenvironment via Stat3/IDO1/AhR axis to boost immunotherapy
Source: Mater Today Bio. 2026 Feb 10;37:102924. doi: 10.1016/j.mtbio.2026.102924 (PMC12930037; doi:10.1016/j.mtbio.2026.102924)
Supplement: Multimedia component 1 [file mmc1.docx]

**Supporting Information**

**Galangin-Loaded Biomimetic Dendritic Cells Membrane Nanovaccine Reprograms the Ovarian Cancer Microenvironment *via* Stat3/IDO1/AhR Axis to Boost Immunotherapy**

Nuerbiye Aobulikasimu ^a, 1,^ ^*^, Lele Fang ^a, 1^, Aidiresi Maimaitiyiming ^b^, Dilimureti Kasimu ^a^, Adila Aipire ^a^, Weilan Wang ^a^, Zhongxiong Fan ^c^, and Jinyao Li ^a, *^

^a^ Xinjiang Key Laboratory of Biological Resources and Genetic Engineering, College of Life Science and Technology, Xinjiang University, Urumqi 830017, China.

^b^ The First Affiliated Hospital of Xinjiang Medical University, Urumqi 830017, China.

^c^ School of Pharmaceutical Sciences, Institute of Materia Medica, Xinjiang University, Urumqi 830017, China

^1^ The two authors contribute equally

**^*^Correspondence:**

E-mail addresses: [nuerbiye@xju.edu.cn](mailto:nuerbiye@xju.edu.cn) (Nuerbiye Aobulikasimu), [ljyxju@xju.edu.cn](mailto:ljyxju@xju.edu.cn) (Jinyao Li)

**Table S1.** The list of Flow-cytometry antibodies

**Table S2.** The primary antibodies and secondary antibodies for Western Blot

**Figure S1.** The Optimization of the Preparation of GA-NPs@DCV.

**Figure S2.** The CETSA melting curve of GA binding to STAT3.

**Figure S3.** TCGA database to investigate the expression levels of IDO1 in ovarian cancer patients

**Figure S4.** Cell viability of GA on the ovarian cancer cells^.^

**Figure S5.** The effect of ID8 cells membranes on the maturation of BMDCs.

**Figure S6.** Cell viability of GA-NPs@DCV and uptake by ID8 cell.

**Figure S7.** The influence of GA-NPs@DCV on T-cell immune response.

**Figure S8.** Acute toxicity studies of GA-NPs@DCV in healthy mice.

**Figure S9.** The immune stimulation ability of a different antigen-carrying NPs@DCV in lymph nodes and tumor in melanoma-bearing mice.

**Figure S10.** GA-NPs@DCV affects the polarization of macrophages in spleen in ovarian cancer bearing mice.

**Figure S11.** Safety evaluation of GA-NPs@DCV in ovarian cancer bearing mice.

**Table S1.** The list of Flow-cytometry antibodies

| **Antibody** | **Dilution** | **Reactivity** | **Clone** | **Manufacturer** | **Citations** |
| --- | --- | --- | --- | --- | --- |
| [PE/Cyanine7 Anti-Mouse CD11c Antibody](https://www.elabscience.cn/p-pe_cyanine7_anti_mouse_cd11c_antibody_n418-e_ab_f0991h) | 1:100 | Mouse | [N418](https://www.elabscience.cn/search-category=%E6%B5%81%E5%BC%8F%E6%8A%97%E4%BD%93&keywords=N418) | Elabscience | [1] |
| [APC Anti-Mouse CD40 Antibody](https://www.elabscience.cn/p-apc_anti_mouse_cd40_antibody_fgk4_5_fgk45-e_ab_f1028e) | 1:50 | Mouse | FGK4.5/FGK45 | Elabscience | [2] |
| FITC Anti-Mouse MHC II (I-A/I-E) Antibody | 1:50 | Mouse | M5/114 | Elabscience | [3] |
| [PE Anti-Mouse CD86 Antibody](https://www.elabscience.cn/p-pe_anti_mouse_cd86_antibody_gl_1-e_ab_f0994d) | 1:50 | Mouse | GL-1 | Elabscience | [4] |
| PE Anti-Mouse/Human CD11b Antibody | 1:100 | Mouse | [M1/70](https://www.elabscience.cn/search-category=%E6%B5%81%E5%BC%8F%E6%8A%97%E4%BD%93&keywords=M1/70) | Elabscience | [5] |
| PE/Cyanine7 Anti-Mouse CD86 Antibody | 1:100 | Mouse | [GL-1](https://www.elabscience.cn/search-category=%E6%B5%81%E5%BC%8F%E6%8A%97%E4%BD%93&keywords=GL-1) | Elabscience | [2] |
| Elab Fluor® Red 780 Anti-Mouse F4/80 Antibody | 1:100 | Mouse | [CI/A3-1](https://www.elabscience.cn/search-category=%E6%B5%81%E5%BC%8F%E6%8A%97%E4%BD%93&keywords=CI:A3-1) | Elabscience | [6] |
| APC Anti-Mouse CD206/MMR Antibody | 1:50 | Mouse | C068C2 | Elabscience | [7] |
| CD3 Monoclonal Antibody (17A2), Alexa Fluor™ 700, eBioscience™ | 1:50 | Mouse | 17A2 | Invitrogen | [8] |
| FITC Anti-Mouse CD4 Antibody | 1:50 | Mouse | GK1.5 | Elabscience | [3] |
| PE/Cyanine7 Anti-Mouse CD8a Antibody | 1:50 | Mouse | 53-6.7 | Elabscience | [9] |
| CD25 Monoclonal Antibody (PC61.5), PE-Cyanine7, eBioscience™ | 1:50 | Mouse | PC-61.5.3 | Invitrogen | [10] |
| PerCP/Cyanine5.5 Foxp3 Antibody | 1:50 | Mouse | FJK-16s | Elabscience | [11] |
| CD45 Monoclonal Antibody (2D1), APC-eFluor™ 780, eBioscience™ | 1:100 | Mouse | 2D1 | Invitrogen | [12] |

**Table S2.** The primary antibodies and secondary antibodies for Western Blot

| **Antibody** | **dilution** | **host** | **manufacturer** | **Citations** |
| --- | --- | --- | --- | --- |
| Ah Receptor Monoclonal Antibody (A-3): sc-133088 | 1:1000 | Mouse | [Santa Cruz Biotechnology](https://www.so.com/link?m=zamKsjv3aGkFDRRS9Wy+CHJkX76onIW815Dhd2nYn3zKT2udJqc91CrF9+/SpPQDX/3P8DhdRTTnW9Bm7kv5Kx6UIWPwLWweeIIjg+/ZbzCY4OfmiTUNpmMZPLNS6K7aLmiE3QooqvjjWMmXzd4ec/Tybg6MYE7TUrU5d9aJkZo6GIPK0wG0czNaFZ0GI6tmu) | [12] |
| IDO1 Monoclonal Antibody (mIDO-48):sc-53978 | 1:1000 | Rat | [Santa Cruz Biotechnology](https://www.so.com/link?m=zamKsjv3aGkFDRRS9Wy+CHJkX76onIW815Dhd2nYn3zKT2udJqc91CrF9+/SpPQDX/3P8DhdRTTnW9Bm7kv5Kx6UIWPwLWweeIIjg+/ZbzCY4OfmiTUNpmMZPLNS6K7aLmiE3QooqvjjWMmXzd4ec/Tybg6MYE7TUrU5d9aJkZo6GIPK0wG0czNaFZ0GI6tmu) | [13] |
| Phospho-STAT3 (Tyr705) Antibody: AF3293 | 1:1000 | Rabbit | Affinity Biosciences | [14] |
| JAK1 Monoclonal Antibody (A-9):sc-1677 | 1:1000 | Mouse | [Santa Cruz Biotechnology](https://www.so.com/link?m=zamKsjv3aGkFDRRS9Wy+CHJkX76onIW815Dhd2nYn3zKT2udJqc91CrF9+/SpPQDX/3P8DhdRTTnW9Bm7kv5Kx6UIWPwLWweeIIjg+/ZbzCY4OfmiTUNpmMZPLNS6K7aLmiE3QooqvjjWMmXzd4ec/Tybg6MYE7TUrU5d9aJkZo6GIPK0wG0czNaFZ0GI6tmu) | [15] |
| Stat3 Antibody: AF6294 | 1:1000 | Rabbit | Affinity Biosciences | [16] |
| Phospho-Jak1(Tyr1034/1035) (D7N4Z) Rabbit mAb #74129 | 1:1000 | Rabbit | Cell Signaling Technology | [17] |
| GAPDH (14C10) Rabbit mAb #2118 | 1:3000 | Rabbit | Cell Signaling Technology | [18] |
| Goat Anti-Mouse IgG | 1:3000 | Goat | Elabscience | [19] |
| Goat Anti-Rabbit IgG | 1:3000 | Rabbit | Elabscience | [20] |

**
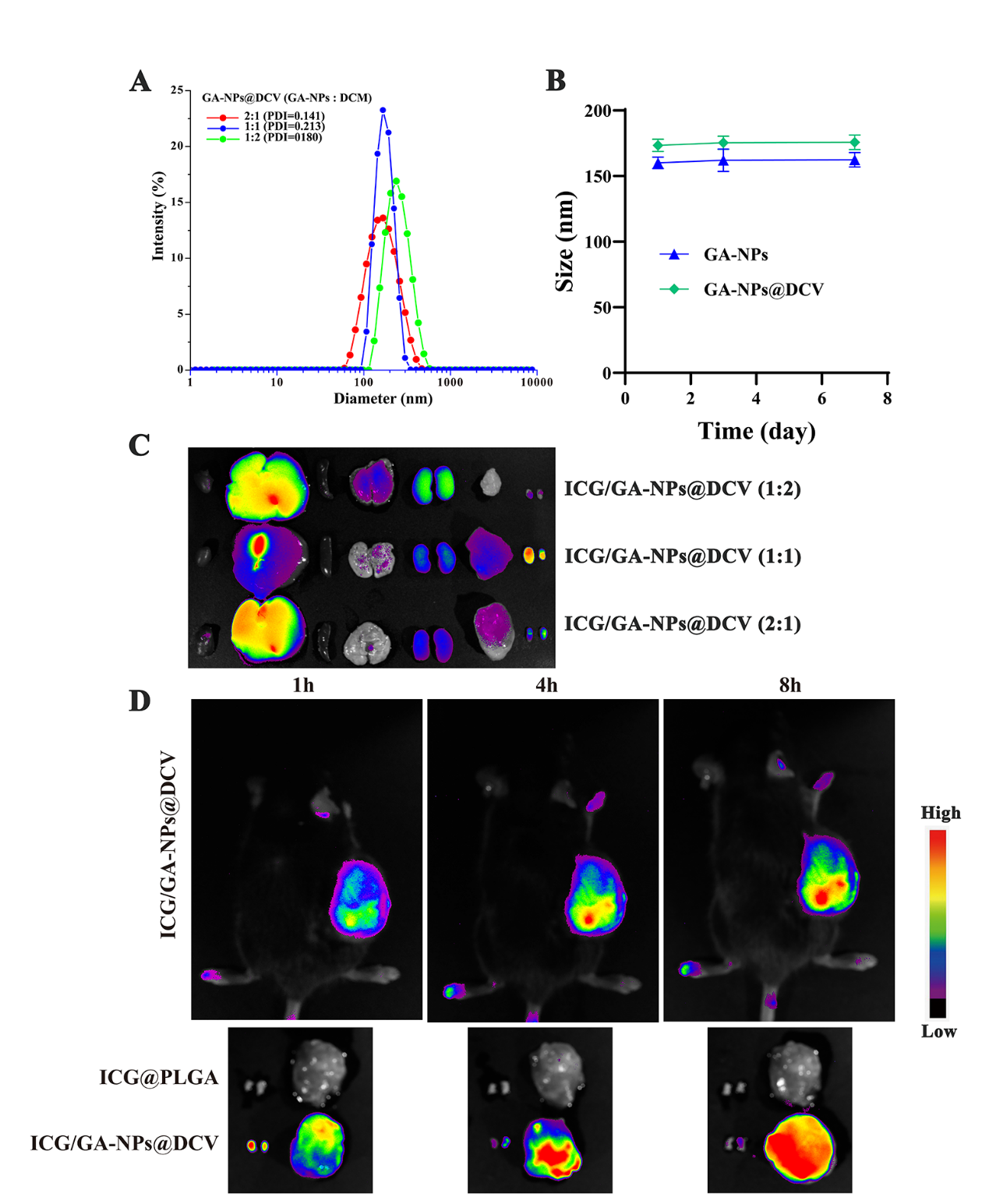
Fig. S1** The Optimization of the Preparation of GA-NPs@DCV. A) The size of GA-NPs@DCV in the different ratio of DCM to GA-NPs. B) The stability of GA-NPs@DCV and GA-NPs. C, D) The accumulation of GA-NPs@DCV in lymph nodes and tumor.

**
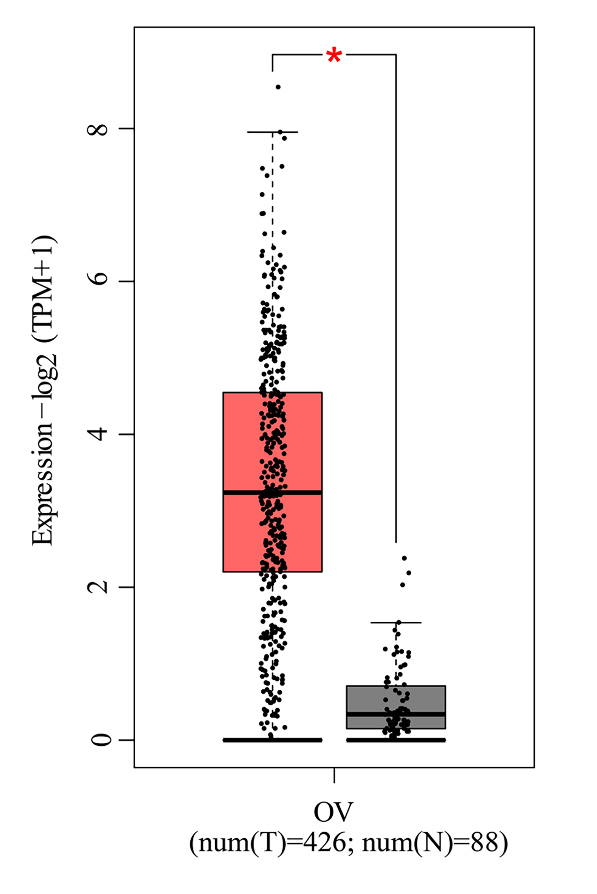
**
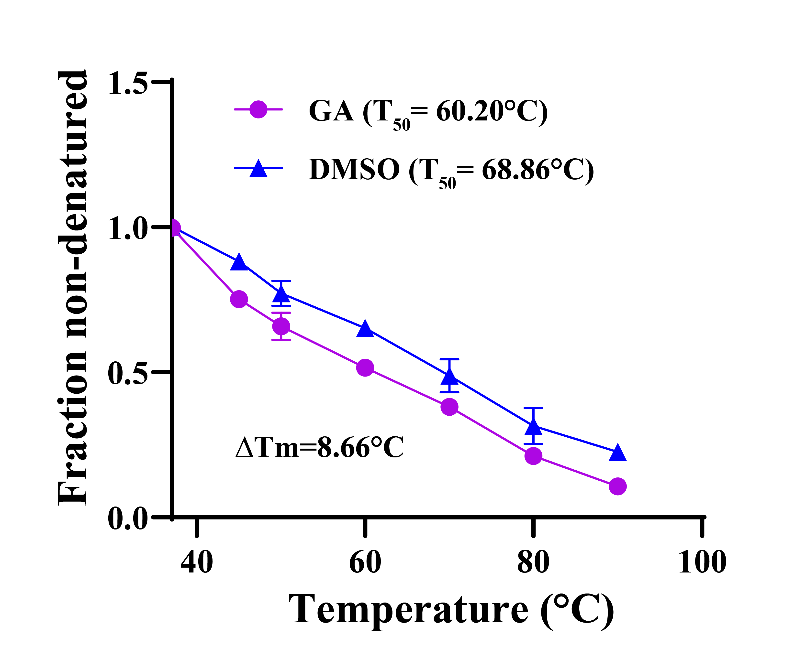
**Fig. S2** The CETSA melting curve of GA binding to STAT3.

**Fig. S3** TCGA database to investigate the expression levels of IDO1 in ovarian cancer patients (**p*<0.05)


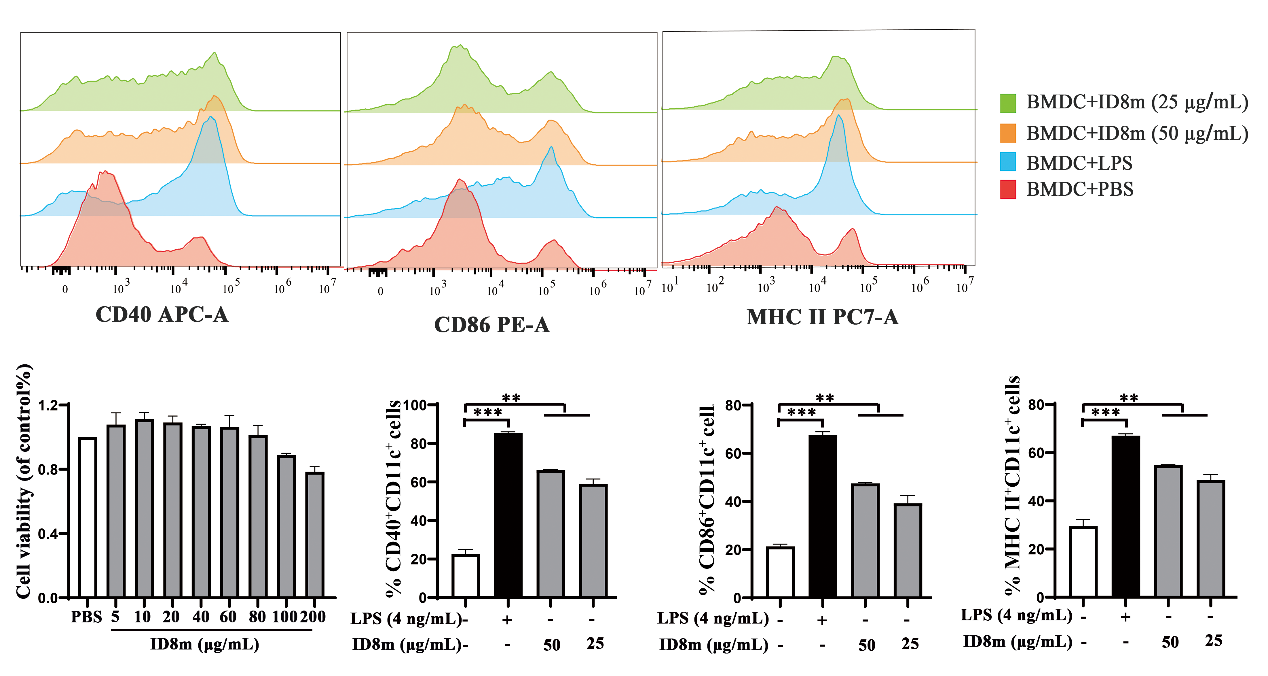
**Fig. S4** Cell viability of GA on the ovarian cancer cells. (^*^*p* < 0.05, ^**^ *p* < 0.01, ^***^ *p* < **
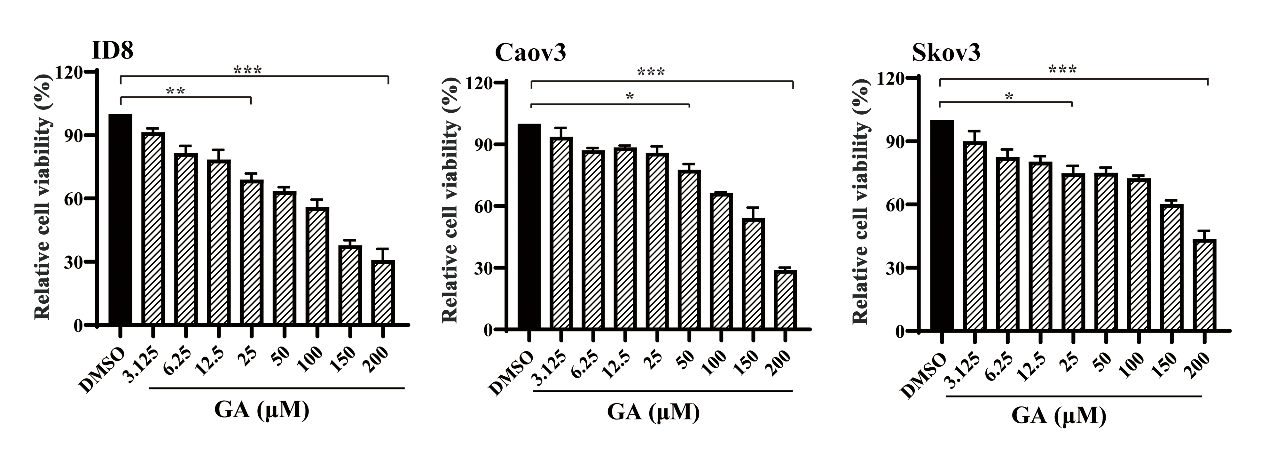
**0.001)

**Fig. S5** The effect of ID8 cells membranes on the maturation of BMDCs. Representative flow cytometry histograms and percentage of CD40^+^, CD86^+^, MHC II^+^ on BMDC after incubation with different concentrations of ID8 cells membranes for 12 h. (^*^*p* < 0.05, ^**^ *p* < 0.01, ^***^ *p* < 0.001)


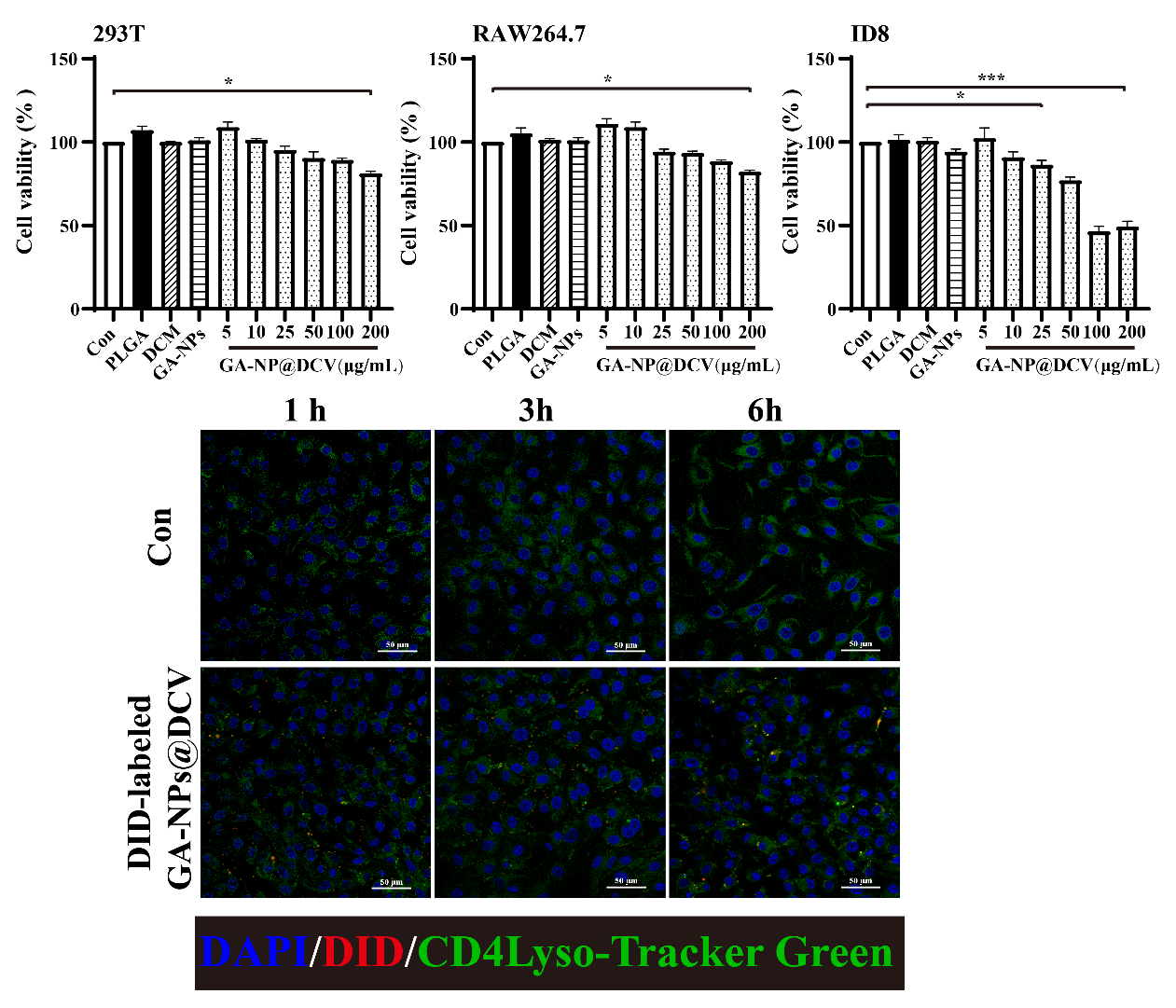
**Fig. S6** Cell viability of GA-NPs@DCV and uptake by ID8 cell. (^*^*p* < 0.05, ^**^ *p* < 0.01, ^***^ *p* < 0.001)


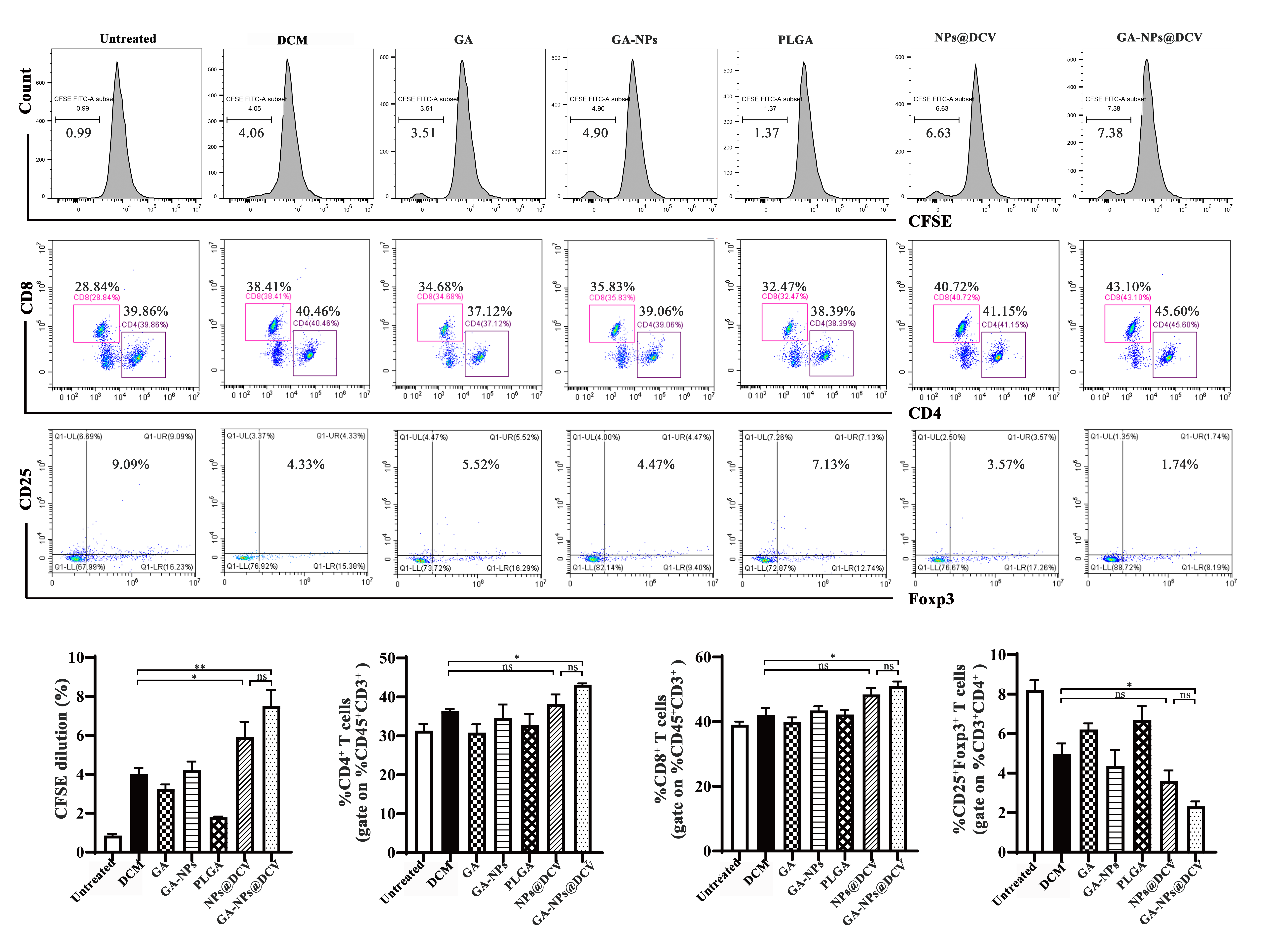
**Fig. S7** The influence of GA-NPs@DCV on T-cell immune response. Representative cytometric analysis and percentage of CD3^+^CD4^+^, CD3^+^CD8^+^, and Treg cells (CD4^+^Foxp3^+^CD25^+^) in splenic lymphocytes cultured with various formulations for 72 h. CFSE dilution was used for quantification of T-cell proliferation. (^*^*p* < 0.05, ^**^ *p* < 0.01, ^***^ *p* < 0.001)

**Fig. S8** Acute toxicity studies of GA-NPs@DCV in healthy mice. A) Body weight changes of tumor-bearing mice during treatment (GA-NPs@DCV represent low, medium, and high dose, respectively 20, 50, 100 mg/Kg). B-F) The serum levels of ALT, AST, Ser, BUN, and CKMB after treatment with the different formations in ovarian cancer bearing mice. J) Representative H&E staining images of heart, liver, spleen, lung, kidney, and ovary from mice treated with different formations. Scale bar = 100 µm.
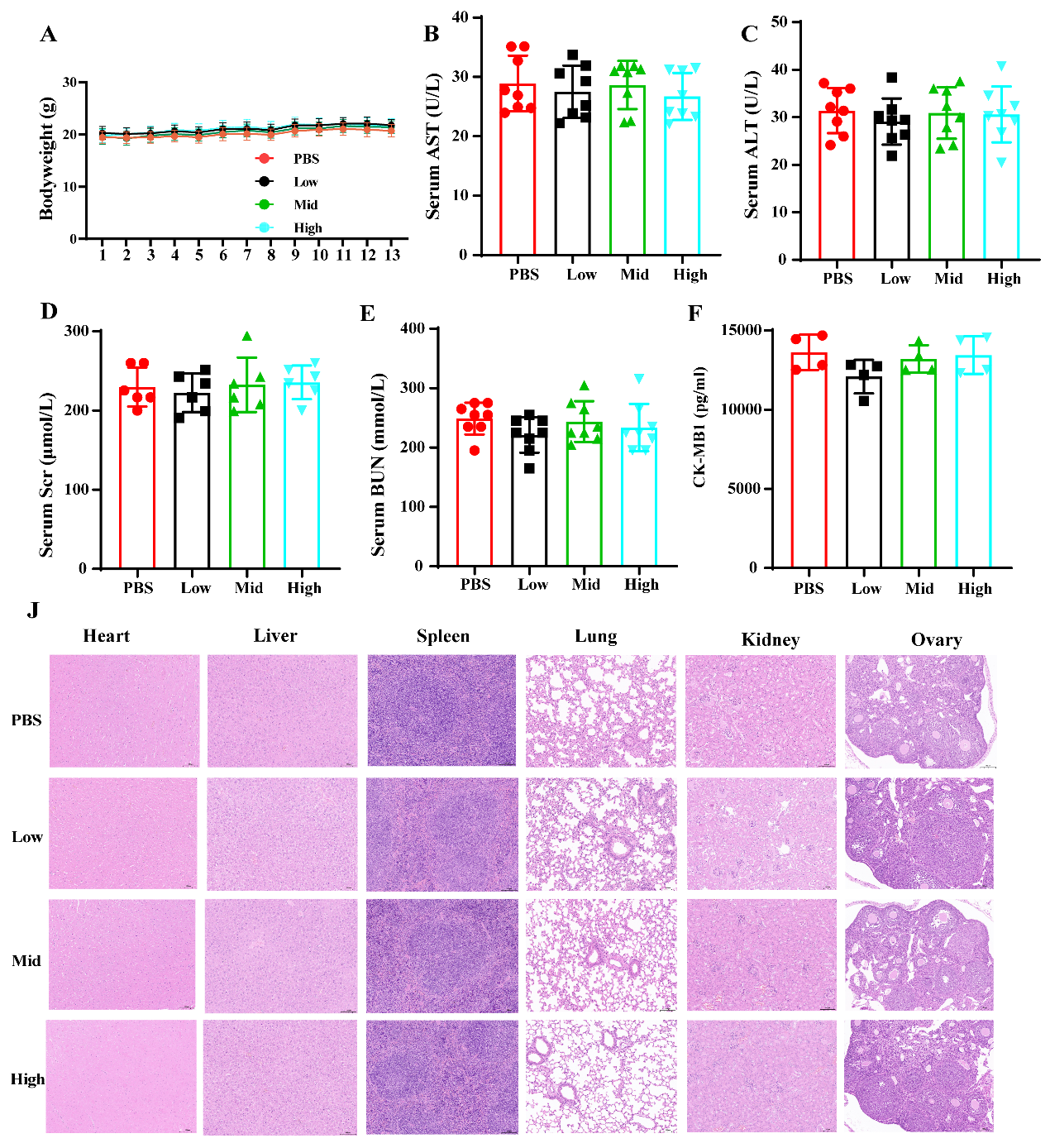


**
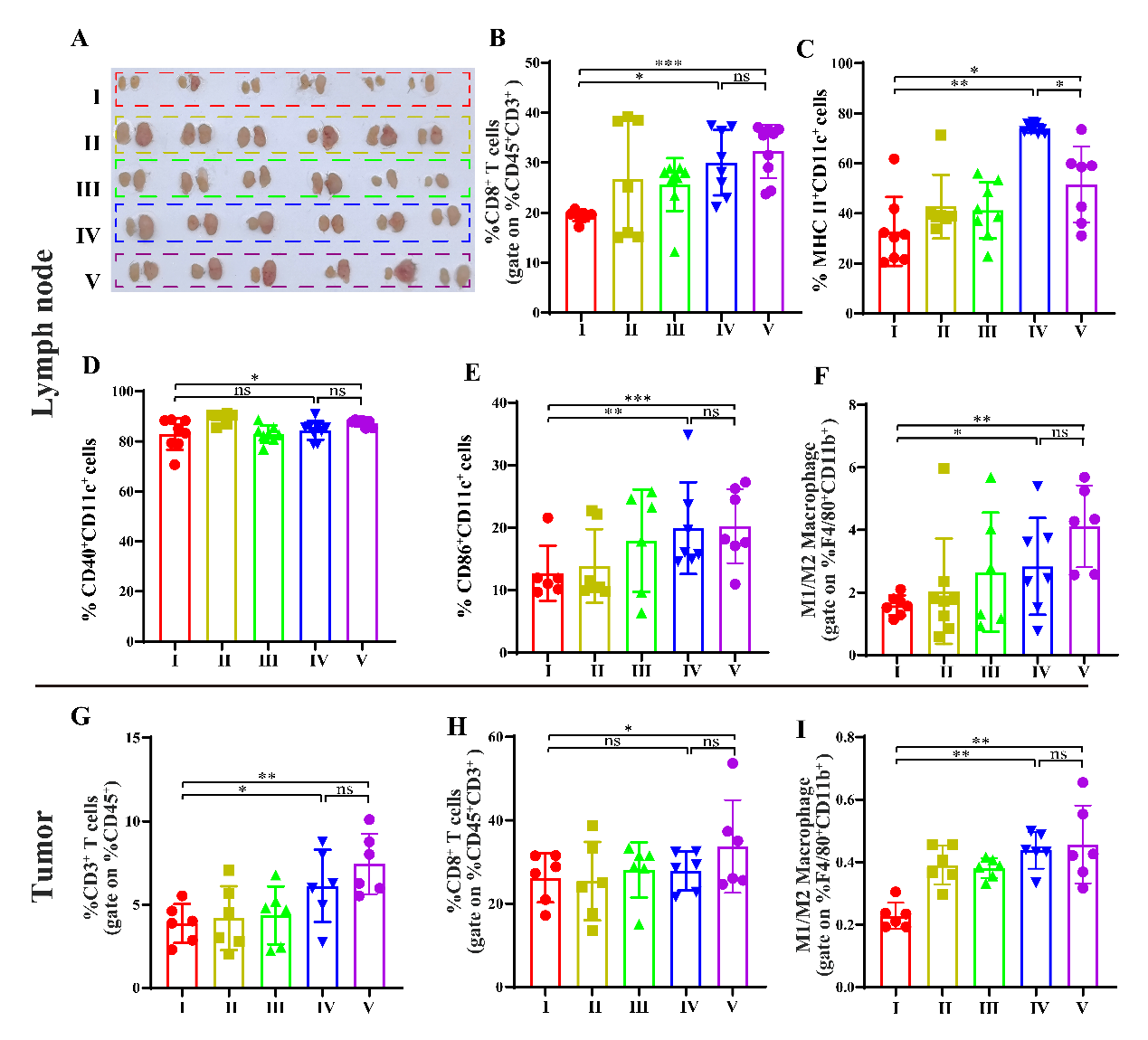
Fig. S9** The immune stimulation ability of a different antigen-carrying NPs@DCV in lymph nodes and tumor in melanoma-bearing mice. The percentage of CD3^+^CD8^+^, M1-type (CD11b^+^CD86^+^ cells) and M2-type (CD11b^+^CD206^+^ cells) macrophages, D40^+^, CD86^+^, MHC II^+^, were detected by flow cytometry in lymph nodes each group of melanoma-bearing mice. (^*^*p* < 0.05, ^**^ *p* < 0.01, ^***^ *p* < 0.001)


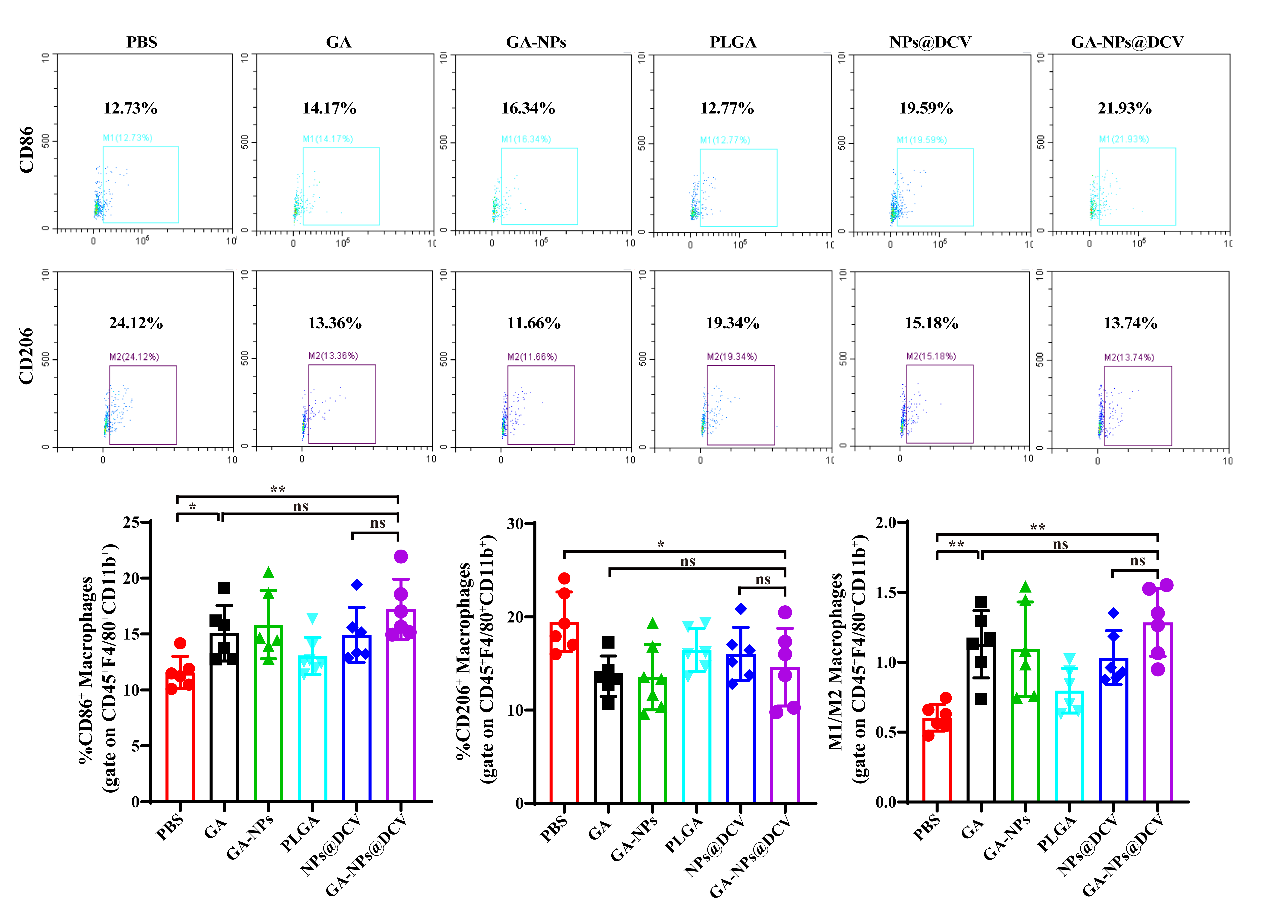
**Fig. S10** GA-NPs@DCV affects the polarization of macrophages in spleen in ovarian cancer bearing mice. The percentage of CD11b^+^CD86^+^, CD11b^+^, CD206^+^ macrophages were detected by flow cytometry in spleen each group of ovarian cancer bearing mice. (^*^*p* < 0.05, ^**^ *p* < 0.01, ^***^ *p* < 0.001)

**
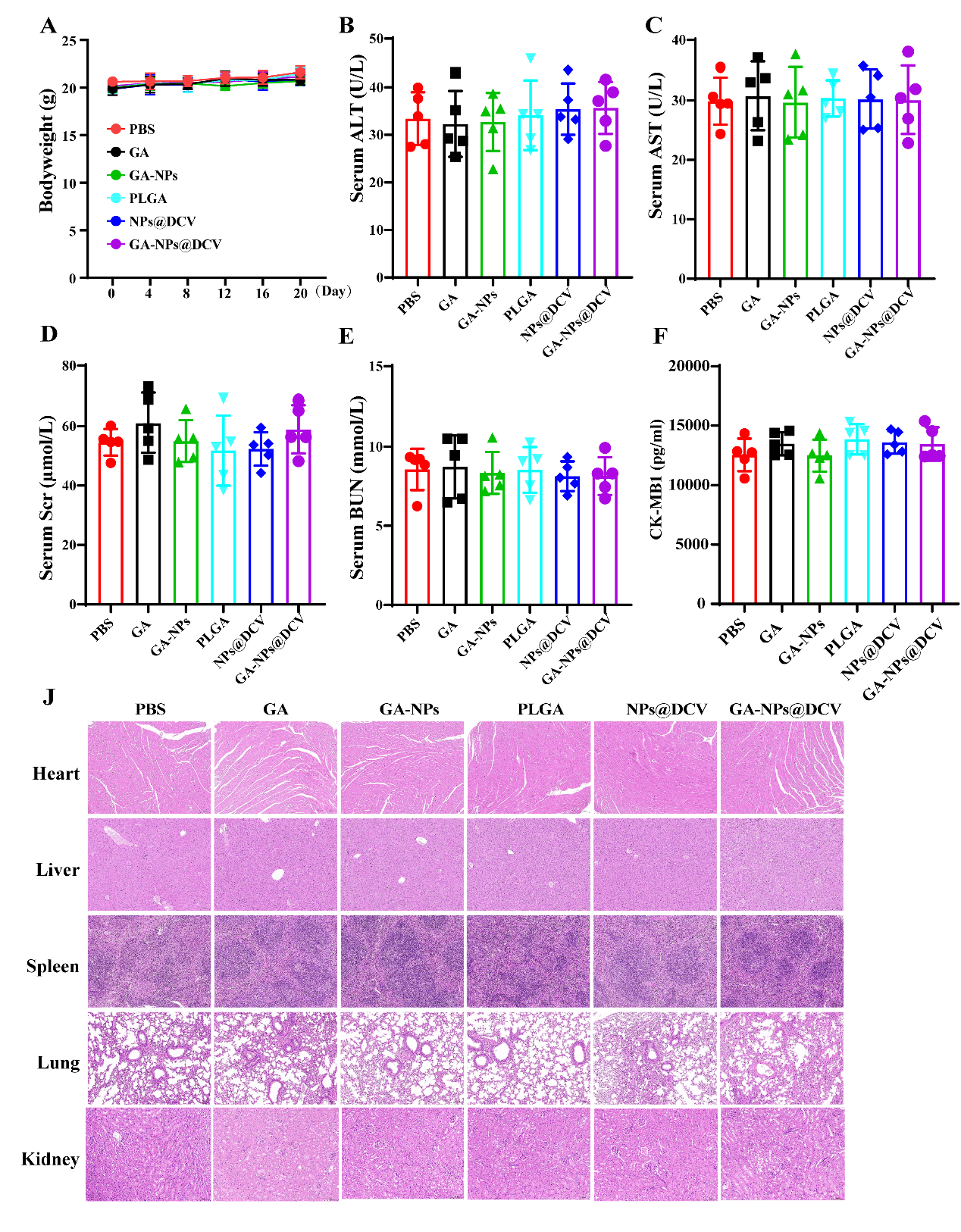
****Fig. S11** Safety evaluation of GA-NPs@DCV in ovarian cancer bearing mice. A) Body weight changes of tumor-bearing mice during treatment. B-F) The serum levels of ALT, AST, Ser, BUN, and CKMB after treatment with the different formations in ovarian cancer bearing mice. J) Representative H&E staining images of heart, liver, spleen, lung, and kidney from mice treated with different formations. Scale bar = 100 µm.

# References

1. Zhou Z, Guo F, Zhang J, Liao L, Jiang M, Huang Y, Liu Y, Lei L, Tao Z, Yu C-Y, Wei H: Facile integration of a binary nano-prodrug with αPD-L1 as a translatable technology for potent immunotherapy of TNBC. *Acta Biomaterialia* 2025, 194:373-384.

2. Feng K, Zhang X, Li J, Han M, Wang J, Chen F, Yi Z, Di L, Wang R: Neoantigens combined with in situ cancer vaccination induce personalized immunity and reshape the tumor microenvironment. *Nature Communications* 2025, 16:5074.

3. Cui Z, Wang H, Qin L, Yuan Y, Xue J, An Y, Sun L, Zhu R, Li Q, Wang Y, et al: Probing the Structural Elements of Polysaccharide Adjuvants for Enhancing Respiratory Mucosal Response: From Surmounting Multi-Obstacles to Eliciting Cascade Immunity. *ACS Nano* 2025, 19:11012-11028.

4. Huang H, Xiao L, Fang L, Lei M, Liu Z, Gao S, Lei Q, Lei J, Wei R, Lei Y, et al: Static Topographical Cue Combined with Dynamic Fluid Stimulation Enhances the Macrophage Extracellular Vesicle Yield and Therapeutic Potential for Bone Defects. *ACS Nano* 2025, 19:8667-8691.

5. Wu Y, Diao P, Peng Y, Yang Y, Wang Y, Lv P, Li J, Wang D, Cai T, Cheng J: A Hybrid Manganese Nanoparticle Simultaneously Eliminates Cancer Stem Cells and Activates STING Pathway to Potentiate Cancer Immunotherapy. *ACS Nano* 2025, 19:12237-12252.

6. Chen H, Wu L, Wang T, Zhang F, Song J, Fu J, Kong X, Shi J: PTT/ PDT-induced microbial apoptosis and wound healing depend on immune activation and macrophage phenotype transformation. *Acta Biomaterialia* 2023, 167:489-505.

7. Tang C, Wang H, Guo L, Cui Y, Zou C, Hu J, Zhang H, Yang G, Zhou W: Multifunctional Nanomedicine for Targeted Atherosclerosis Therapy: Activating Plaque Clearance Cascade and Suppressing Inflammation. *ACS Nano* 2025, 19:3339-3361.

8. Wiernicki B, Maschalidi S, Pinney J, Adjemian S, Vanden Berghe T, Ravichandran KS, Vandenabeele P: Cancer cells dying from ferroptosis impede dendritic cell-mediated anti-tumor immunity. *Nature Communications* 2022, 13:3676.

9. Zheng X, Liu Y, Liu Y, Zang J, Wang K, Yang Z, Chen N, Sun J, Huang L, Li Y, et al: Arginine-assembly as NO nano-donor prevents the negative feedback of macrophage repolarization by mitochondrial dysfunction for cancer immunotherapy. *Biomaterials* 2024, 306:122474.

10. Yao RQ, Li ZX, Wang LX, Li YX, Zheng LY, Dong N, Wu Y, Xia Z-F, Billiar TR, Ren C, Yao Y-M: Single-cell transcriptome profiling of the immune space-time landscape reveals dendritic cell regulatory program in polymicrobial sepsis. *Theranostics* 2022, 12:4606-4628.

11. Li T, Liu X, Han P, Aimaier A, Zhang Y, Li J: Syringaldehyde ameliorates mouse arthritis by inhibiting dendritic cell maturation and proinflammatory cytokine secretion. *International Immunopharmacology* 2023, 121:110490.

12. Huang Y, Happonen KE, Burrola PG, O'Connor C, Hah N, Huang L, Nimmerjahn A, Lemke G: Microglia use TAM receptors to detect and engulf amyloid β plaques. *Nature Immunology* 2021, 22:586-594.

13. Xu X, Zhang Y, Lu Y, Zhang X, Zhao C, Wang J, Guan Q, Feng Y, Gao M, Yu J, et al: CD58 Alterations Govern Antitumor Immune Responses by Inducing PDL1 and IDO in Diffuse Large B-Cell Lymphoma. *Cancer Research* 2024, 84:2123-2140.

14. Wang J, Wang Y, Xiaohalati X, Su Q, Liu J, Cai B, Yang W, Wang Z, Wang L: A Bioinspired Manganese-Organic Framework Ameliorates Ischemic Stroke through its Intrinsic Nanozyme Activity and Upregulating Endogenous Antioxidant Enzymes. *Advanced Science (Weinheim, Baden-Wurttemberg, Germany)* 2023, 10:e2206854.

15. Runtsch MC, Angiari S, Hooftman A, Wadhwa R, Zhang Y, Zheng Y, Spina JS, Ruzek MC, Argiriadi MA, McGettrick AF, et al: Itaconate and itaconate derivatives target JAK1 to suppress alternative activation of macrophages. *Cell Metabolism* 2022, 34.

16. Xie J, Wu X, Zheng S, Lin K, Su J: Aligned electrospun poly(L-lactide) nanofibers facilitate wound healing by inhibiting macrophage M1 polarization via the JAK-STAT and NF-κB pathways. *Journal of Nanobiotechnology* 2022, 20:342.

17. Lu L, Xu K, Qu H, Song L, Song Y, Wu Y, Sun X, Kong J, Wen Q, Jiao J, et al: AF6 regulates intestinal IgA via crosstalk between intestinal epithelial cells and immune cells in inflammatory bowel disease. *IScience* 2025, 28:112658.

18. Silvestro S, Calabrò M, Trainito A, Salamone S, Pollastro F, Mazzon E, Minuti A: Cannabinol's Modulation of Genes Involved in Oxidative Stress Response and Neuronal Plasticity: A Transcriptomic Analysis. *Antioxidants (Basel, Switzerland)* 2025, 14.

19. Zhang X, Zhang Z, Zhao Y, Jin L, Tai Y, Tang Y, Geng S, Zhang H, Zhai Y, Yang Y, et al: Sodium chloride promotes macrophage pyroptosis and aggravates rheumatoid arthritis by activating SGK1 through GABA receptors Slc6a12. *International Journal of Biological Sciences* 2024, 20:2922-2942.

20. Yang M, Cui W, Lv X, Xiong G, Sun C, Xuan H, Ma W, Cui X, Cheng Y, Han L, Chu B: S100P is a ferroptosis suppressor to facilitate hepatocellular carcinoma development by rewiring lipid metabolism. *Nature Communications* 2025, 16:509.
